# Inactivation of JNK signalling results in polarity loss and cell senescence of Sertoli cell

**DOI:** 10.1111/cpr.13760

**Published:** 2024-09-27

**Authors:** Zhiming Shen, Yang Gao, Xuedong Sun, Min Chen, Changhuo Cen, Mengyue Wang, Nan Wang, Bowen Liu, Jiayi Li, Xiuhong Cui, Jian Hou, Yuhua Shi, Fei Gao

**Affiliations:** ^1^ Guangdong Cardiovascular Institute Guangdong Provincial People's Hospital, Guangdong Academy of Medical Sciences Guangzhou China; ^2^ Department of Reproductive Medicine Guangdong Provincial People's Hospital (Guangdong Academy of Medical Sciences), Southern Medical University Guangzhou China; ^3^ State Key Laboratory of Stem Cell and Reproductive Biology, Institute of Zoology Chinese Academy of Sciences Beijing China; ^4^ Institute for Stem Cell and Regeneration Chinese Academy of Sciences Beijing China; ^5^ University of Chinese Academy of Sciences Beijing China; ^6^ Eastern Department of Neurology Guangdong Provincial People's Hospital (Guangdong Academy of Medical Sciences), Southern Medical University Guangzhou China; ^7^ Department of Neurology Nanfang Hospital, Southern Medical University Guangzhou China; ^8^ Beijing Institute for Stem Cell and Regenerative Medicine Beijing China; ^9^ State Key Laboratory of Animal Biotech Breeding, College of Biological Sciences China Agricultural University Beijing China; ^10^ Department of Obstetrics and Gynecology, Center for Reproductive Medicine Nanfang Hospital, Southern Medical University Guangzhou China

## Abstract

As major somatic cells in the testis, Sertoli cell development is precisely regulated by numerous factors, and aberrant development of these cells is associated with male reproductive diseases. JNK signalling is evolutionarily conserved and involved in multiple critical biological processes. Here, we found that the double knockout of *Jnk1* and *Jnk2* resulted in aberrant localisation of Sertoli cells at early developmental stages, with most Sertoli cells being lost at later stages. Further studies revealed that the inactivation of JNK signalling caused polarity loss in Sertoli cells. In vitro‐cultured *Jnk1/2‐DKO* Sertoli cells exhibited a senescence‐associated phenotype. Mechanistic studies demonstrate that JNK signalling is likely involved in establishing Sertoli cell polarity by regulating the expression of TGF‐β2, mediated by *c‐Jun*. The senescence of Sertoli cells in JNKs‐deficient mice is caused by aberrant proteolysis of P27^KIP1^, mediated by *c‐Myc*. This study demonstrates the role of JNK signalling in Sertoli cell development and functional maintenance, which may also represent an aetiology of male infertility in humans.

## Graphical abstract

JNK signalling plays a critical role in Sertoli cells development and functional maintenance. Disruption of JNK signalling leads to polarity loss and cell senescence in Sertoli cells, resulting in aberrant testis development and male infertility in mouse models.

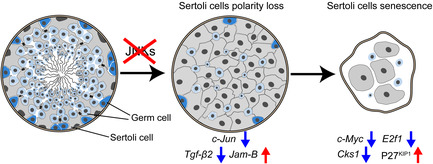

## 1 INTRODUCTION

Sertoli cells are the major supporting cells in the testis, intimately interacting with spermatogenic cells to provide both physical and nutritional support for spermatogenesis. They are the first sex‐specific cell type identified in foetal testes and are involved in the formation of testis cords.^1^ Anti‐Müllerian hormone (AMH) secreted by Sertoli cells induces the regression of the Müllerian ducts in the male mesonephros.^1^, ^2^ It is generally accepted that Sertoli cells undergo terminal differentiation and permanently exit the cell cycle at puberty.^3^, ^4^ Mature Sertoli cells are highly polarised epithelial cells, with their polarity established at ~2 weeks after birth in mouse models. This polarity is characterised by nuclei located in the basal compartment and cytoplasm protruding toward the lumen of seminiferous tubules. Adjacent polarised Sertoli cells form the blood‐testis barrier (BTB) near the basement membrane, which sequesters virtually all events associated with meiotic and post‐meiotic germ cell maturation from systemic circulation.^5^, ^6^ Furthermore, Sertoli cells secrete factors necessary for normal germ cell development and self‐renewal of spermatogonial stem cells, including GDNF, FGF2, WNT family proteins, and retinoic acid (RA).^7^, ^8^

The c‐Jun N‐terminal kinase (JNK) signalling cascade, part of the mitogen‐activated protein kinase (MAPK) family, is evolutionarily conserved and widely recognised for its roles in regulating embryonic development, tissue regeneration, cell proliferation, and apoptosis.^9^ In mammals, the JNKs subfamily consists of three members: *Jnk1*, *Jnk2*, and *Jnk3* (also known as MAPK8, MAPK9, and MAPK10). *Jnk1* and *Jnk2* are expressed in various tissues, whereas *Jnk3* is primarily expressed in the brain and heart.^10^, ^11^, ^12^ The functions of *Jnk1* and *Jnk2* are redundant. *Jnk1* and *Jnk2* single knockout mice are viable, but double knockout mice are embryonic lethal.^13^, ^14^ JNKs regulate gene expression through phosphorylation and activation of activator protein 1 (AP‐1) and AP‐1‐related transcription factors (e.g., *c‐Jun*, *JunB*, *JunD*, and ATF2). Moreover, JNKs modulate AP‐1 activity by inducing the expression of *c‐Jun* (through action at AP‐1‐like binding sites in the *c‐Jun* promoter) and by regulating the half‐life of the c‐Jun protein.^15^, ^16^ It has been reported that JNK signalling is involved in modulating BTB function and germ cell migration. The impact of TNF‐α on the integrity of the BTB is mediated through JNK signalling pathway, as evidenced by its modulation of the expression of Coxsackie‐ and adenovirus‐receptor‐like transmembrane protein (CLMP) and intercellular adhesion factor‐1 (ICAM‐1).^17^, ^18^, ^19^ In a CdCl2‐induced rat model, the disruption of the BTB is mediated by the downregulation of JNK signalling pathway, which induces the expression of α2‐macroglobulin (α2‐MG), a protease inhibitor involved in the dynamic regulation of the BTB.^20^ Despite studies strongly supporting the role of JNK signalling in spermatogenesis, the exact functions and underlying mechanisms are still unclear.

In this study, we employed a conditional gene ablation strategy to specifically inactivate both *Jnk1* and *Jnk2* in Sertoli cells. We found that JNK signalling is involved in Sertoli cell polarity establishment by regulating the expression of TGF‐β2 and *Jam‐B*, which is mediated by *c‐Jun*. Inactivation of JNK signalling leads to the aberrant proteolysis of P27^KIP1^, which in turn causes senescence of Sertoli cells.

## 2 MATERIALS AND METHODS

### 2.1 Mice

All animal experiments were performed in accordance with the regulations of the Institutional Animal Care and Use Committee (IACUC) at the Institute of Zoology, Chinese Academy of Sciences. All mice were maintained on a C57BL/6; 129/SvEv mixed background. *Jnk1* ^*+/flox*^ mice were obtained from the Laboratory Animal Center, Institute of Zoology, Chinese Academy of Sciences. Detailed information on the generation of the *Jnk1* ^*+/flox*^ mice is provided in Figure S1. *Jnk2* ^*+/Δ*^ mice (Strain No. 004321) were obtained from The Jackson Laboratory. *c‐Jun* ^*+/flox*^ mice (Strain No. T007838) and *Amh* ^*+/Cre*^ mice (Strain No. T007700) were purchased from GemPharmatech (Nanjing, China). *Jnk1* ^*flox/flox*^ *;Jnk2* ^*Δ/Δ*^ *;Amh* ^*+/Cre*^ (*Jnk1/2‐DKO*) male mice were obtained by crossing *Jnk1* ^*flox /flox*^ *;Jnk2* ^*Δ/Δ*^ male mice with *Jnk1/2‐DKO* female mice. *Jnk1* ^*flox/flox*^ *;Jnk2* ^*Δ/Δ*^ male mice were used as control. *c‐Jun* ^*flox/flox*^ *;Amh* ^*+/Cre*^ (*c‐Jun‐KO*) male mice were obtained by crossing *c‐Jun* ^*+/flox*^ *;Amh* ^*+/Cre*^ mice with *c‐Jun* ^*flox/flox*^ mice. *c‐Jun* ^*+/flox*^ *;Amh* ^*+/Cre*^ male mice were used as controls. The *Jnk1* ^*flox*^ allele was genotyped using primers 5′‐ACACTCAGTGGATCTTGGGT‐3′ and 5′‐GAAGACGTTTTTGTTCTTTTGCT‐3′ to determine the presence of *Jnk1* ^*flox*^ (530 bp) or WT (450 bp) alleles. *Jnk2* ^*+/Δ*^, *c‐Jun* ^*+/flox*^, and *Amh* ^*+/Cre*^ mice were genotyped using polymerase chain reaction protocols for the specific strains found on The Jackson Laboratory or GemPharmatech websites.

### 2.2 Tissue collection and histological analysis

Testes or epididymides from control and mutant mice were dissected immediately after euthanasia and then fixed with 4% paraformaldehyde (PFA) or Bouin's solution for 24 h. After being stored in 70% ethanol and embedded in paraffin, 5‐μm thick sections were prepared using a rotary microtome (Leica) and mounted on glass slides. After deparaffinisation, Bouin's solution‐fixed sections were stained with haematoxylin and eosin (H&E) following the standard protocol.

### 2.3 Immunostaining and confocal microscopy

Immunohistochemistry (IHC) and immunofluorescence (IF) of paraffin sections were performed as described previously.^21^ For immunofluorescence of frozen sections, testes were washed in PBS three times and fixed in 4% PFA at 4°C for 2 h. After being washed in PBS three times, the testes were dehydrated in 10% and 30% sucrose in PBS overnight at 4°C. The testes were embedded in OCT (Sakura) and stored at −80°C. Next, 10‐μm thick sections were mounted on glass slides and stored at −20°C until use. The slides were dried at 37°C for 1 h and then the OCT was washed out in PBS. The sections were blocked with 5% BSA in PBST (0.3% Triton X‐100 in PBS) for 1 h at room temperature (RT) and then incubated with primary antibodies in PBST containing 1% BSA for 1 h at RT. Next, the sections were washed with PBS and incubated with Alexa‐conjugated secondary antibodies (Jackson ImmunoResearch) for 1 h at RT. After being washed three times in PBS, the nuclei were stained with DAPI. Images were captured with a confocal laser scanning microscope (Leica, TCS SP8). Primary antibodies and dilution ratios are listed in Table S1.

### 2.4 Isolation and culture of Sertoli cells, transient transfection, and luciferase assay

Sertoli cells were isolated from 1‐week‐old mice. The testes were decapsulated under a dissection microscope. The seminiferous tubules were pooled and washed three times with PBS. The tubules were incubated in PBS containing 1 mg/mL collagenase IV (VETEC, V900893) with circular agitation (85 rpm) for 5 min at 37°C. The tubules were allowed to settle and then washed in PBS. A second enzyme digestion was performed in PBS containing 1 mg/mL collagenase IV, 1 mg/mL hyaluronidase (SIGMA, SIALH3506), 0.25% trypsin, and 1 mg/mL DNase I (AppliChem, A37780500) with circular agitation (85 rpm) for 15 min at 37°C. FBS was added to stop the digestion, and the cell suspension was filtered through a 70 μm filter. Cells were centrifuged, washed, and then plated in a 6‐well plate in DMEM/F12 supplemented with 10% FBS. When cells were ~70% confluent, plasmids were transfected into Sertoli cells using Lipofectamine™ 3000 (Invitrogen, L3000015) in accordance with the manufacturer's instructions. At the end of the culture, cells were lysed for qRT‐PCR, western blot analysis, or luciferase activity analysis using a dual luciferase reporter assay system (Promega, E1910).

### 2.5 Cell proliferation assay

Sertoli cells were cultured in DMEM/F12 in 24‐well plates at 1 × 10^4^ cells per well. The relative number and viability of Sertoli cells were evaluated using the 3‐(4,5‐dimethylthiazol‐2‐yl)‐2,5‐diphenyl‐2H‐tetrazolium bromide (MTT) assay on days 1, 2, 3, 4, 5, and 6. In brief, Sertoli cells seeded in 24‐well plates were washed with PBS twice and incubated with 500 μL of 0.5 mg/mL MTT (Solarbio, IM0280) solution for 4 h at 37°C. Then, the medium containing MTT was removed, and 750 μL of DMSO was added. After incubation for 10 min on a shaking table at 75 rpm, the absorbance was measured spectrophotometrically at 490 nm (OD490) using a multifunction enzyme‐linked analyser (BioTek Synergy 4).

### 2.6 Plasmids construction

All the overexpression and luciferase reporter plasmids were constructed in the pEGFP‐N1 vector or the pGL3 basic plasmids using a pEASY‐Uni Seamless Cloning and Assembly Kit (TransGen). Point mutations were generated using the Fast Mutagenesis System (TransGen). cDNA was amplified from adult wild‐type mouse testes by RT‐PCR. The promoter fragments of *c‐Jun*, *Jam‐B*, or TGF‐β2 were amplified by PCR using the KOD One™ PCR Master Mix (TOYOBO, KMM‐101). All constructs were verified by sequencing. The primers used for constructing the plasmids are listed in Table S2.

### 2.7 Western blot analysis

Isolated Sertoli cells were washed with cold PBS and lysed with RIPA buffer (50 mM Tris–HCl [pH 7.5], 150 mM NaCl, 1% NP‐40, 0.1% SDS, 1% sodium deoxycholate, 5 mM EDTA) supplemented with protease inhibitor cocktail, phosphatase inhibitor cocktail (Roche) and 1 mM PMSF. Equal amounts of total protein were resolved by SDS‐PAGE gels, transferred onto a nitrocellulose membrane, and blocked with 5% fat‐free milk diluted with ×1 TBST (0.05% Tween‐20 in TBS) for 1 h at RT. After washing three times with TBST, the nitrocellulose membranes were probed with primary antibodies overnight at 4°C. After washing three times with TBST buffer, the nitrocellulose membranes were incubated with fluorochrome‐conjugated secondary antibodies in the dark for 1 h at RT. All images were captured using the Odyssey CLx Imaging System (LI‐COR Biosciences). Densitometry was performed using ImageJ software (version 1.52a). GAPDH was used as an internal control to correct for differences in protein loading. Protein signals were quantitatively analysed by comparing the signal intensity of target proteins to internal controls to evaluate their expression levels. The blots are representative of three independent experiments. The primary antibodies used are listed in Table S1.

### 2.8 Quantitative reverse transcription‐polymerase chain reaction (qRT‐PCR) analysis

Total RNA was extracted from isolated Sertoli cells using a RNeasy Kit (Aidlab, RN28) following the manufacturer's instructions. cDNAs were synthesised using ×5 All‐In‐One RT MasterMix (Abm, G592) with 2 μg of total RNA, following the manufacturer's instructions. The resulting cDNAs were used as templates for amplification under the following PCR conditions: 95°C for 2 min, followed by 40 cycles of 95°C for 15 s and 60°C for 30 s. Quantitative PCR assays were performed using ×2 SYBR Green qPCR Mix (Aidlab, PC5902) on a Roche LightCycler 480 II. All primers used in our study were designed to span exon–exon junctions, and the GAPDH gene was used as a housekeeping gene to normalise target gene expression. Sample ΔCt values were calculated as the difference between the Ct values of the target genes and GAPDH. Sample ΔΔCt values were calculated as the difference in ΔCt values between the sample and the control. Relative gene expression levels were determined using 2^−ΔΔCt^ equation. The primers used for qRT‐PCR are listed in Table S3.

### 2.9 RNA‐sequencing analysis

Total RNA was extracted from isolated control and *Jnk1/2‐DKO* Sertoli cells and then sent to Annoroad Gene Technology Corporation (Beijing, China) for RNA library construction and sequencing. The sequenced raw data were filtered to remove adaptor reads, low‐quality reads, and reads with a single copy number. Clean reads were classified according to their copy number, and the saturation of the library was analysed. All aligned RNA‐Seq reads were mapped to GRCm38 using HISAT2 software, and the Fragments Per Kilobase of exon model per Million mapped reads (FPKM) of each gene were calculated using Cufflinks. Differentially expressed genes (FDR <0.05, log_2_[fold change] ≥1 or ≤−1) between control and *Jnk1/2‐DKO* Sertoli cells were identified using DESeq2.^22^ The differentially expressed genes were analysed using Gene Ontology (GO) and Kyoto Encyclopedia of Genes and Genomes (KEGG) analysis by g:Profiler version e105. Heatmaps of the differentially expressed genes were drawn using ImageGP (http://www.ehbio.com/Cloud_Platform/front).

### 2.10 Comet assay

Single‐cell nuclear DNA damage was measured using DNA damage Assay Kit (Nanjing Jiancheng Bioengineering Institute, G010‐1‐1). Control and *Jnk1/2‐DKO* Sertoli cells were suspended in low melting point agarose (0.5%) in PBS at 37°C and spread onto glass slides. After lysing the cellular membranes in Lysis Buffer at 4°C for 2 h, slides were placed in a horizontal gel electrophoresis chamber and incubated in alkaline electrophoresis buffer (1 mM EDTA, 300 mM NaOH) for 30 min. Electrophoresis was performed for 30 min at 25 V. Afterwards, the slides were washed with neutralisation buffer and distilled H_2_O. The gels were stained with propidium iodide and analysed using a fluorescence microscope (Nikon ECLIPSE Ni). Comet parameters of at least 200 cells per sample were analysed using the OpenComet_imagej_v1.3.1 software.

### 2.11 Biotin tracer

Four‐week‐old control and *Jnk1/2‐DKO* male mice were anaesthetised with Avertin, and 20 μL of 10 mg/mL biotin (EZ‐Link Sulfo‐NHS‐LC‐Biotin, Thermo Fisher, A39257) freshly diluted in PBS containing 1 mM CaCl_2_ was injected into the interstitium of one testis, while the other testis was injected with 20 μL of 1 mM CaCl_2_ in PBS as an internal control. The mice were euthanised 30 min later, and the testes were harvested and embedded in OCT. To detect biotin binding in testicular cryosections, FITC‐conjugated streptavidin (Solarbio, SF068) was applied for 1 h at RT, along with DAPI to stain the nuclei. The sections were examined using a confocal laser scanning microscope (Leica, TCS SP8).

### 2.12 SA‐β‐Gal staining

Cultured Sertoli cells were washed in PBS and fixed at RT for 5 min in 2% formaldehyde and 0.2% glutaraldehyde. The fixed cells were stained with fresh staining solution (40 mM citric acid/Na phosphate buffer, 5 mM K_4_[Fe(CN)_6_], 5 mM K_3_[Fe(CN)_6_], 150 mM NaCl, 2 mM MgCl_2_, 1 mg/mL X‐gal) at 37°C overnight. After washing in PBS, images were captured using a microscope digital camera (Nikon DS‐Ri2). For quantitative analysis, at least six random fields from three independent samples in three separate experiments were counted.

### 2.13 Statistical analysis

All experiments were repeated at least three times. Three to five individual animals of each genotype at each time point were performed. For quantitative analysis, at least 30 cross‐sections of seminiferous tubules from six testicular sections per mouse were counted. The quantitative results are presented as the mean ± SEM. The data were analysed using GraphPad Prism version 9.0.0. Unpaired two‐tailed Student's *t*‐tests were used for comparison between two groups. For three or more groups, data were analysed using one‐way ANOVA. The *p*‐values<0.05 were considered to indicate significance.

## 3 RESULTS

### 3.1 Inactivation of JNK signalling in Sertoli cells causes aberrant testicular development and male infertility

To investigate the roles of JNK signalling in spermatogenesis, we initially examined the expression of JNKs in the testes through immunostaining. As shown in Figure S1A, JNKs were predominantly localised in the cytoplasm of WT1‐positive Sertoli cells (a, b, white arrows), suggesting their potential role in Sertoli cell development. To evaluate the functions of JNK signalling in Sertoli cell development, we generated *Jnk1* ^*flox*^ mice using CRISPR‐Cas9 technology, where exon 3 of *Jnk1* was flanked by two *lox*P sites (Figure S1B). By crossing *Jnk1* ^*flox*^ mice with *Jnk2* ^*Δ/Δ*^ and *Amh* ^*+/Cre*^ mice, we obtained *Jnk1/2‐DKO* mice (Figure S1C). In this mouse model, *Jnk1* and *Jnk2* were specifically inactivated in Sertoli cells by *Amh‐Cre*, which was activated in Sertoli cells at approximately E14.5.^23^ qRT‐PCR results showed a dramatic reduction in *Jnk1* mRNA levels in Sertoli cells at P7 (Figure S1D). The reduced expression of JNKs in the Sertoli cells of *Jnk1/2‐DKO* mice was further confirmed by immunofluorescence and western blotting (Figure S1A,E).

**FIGURE 1:**
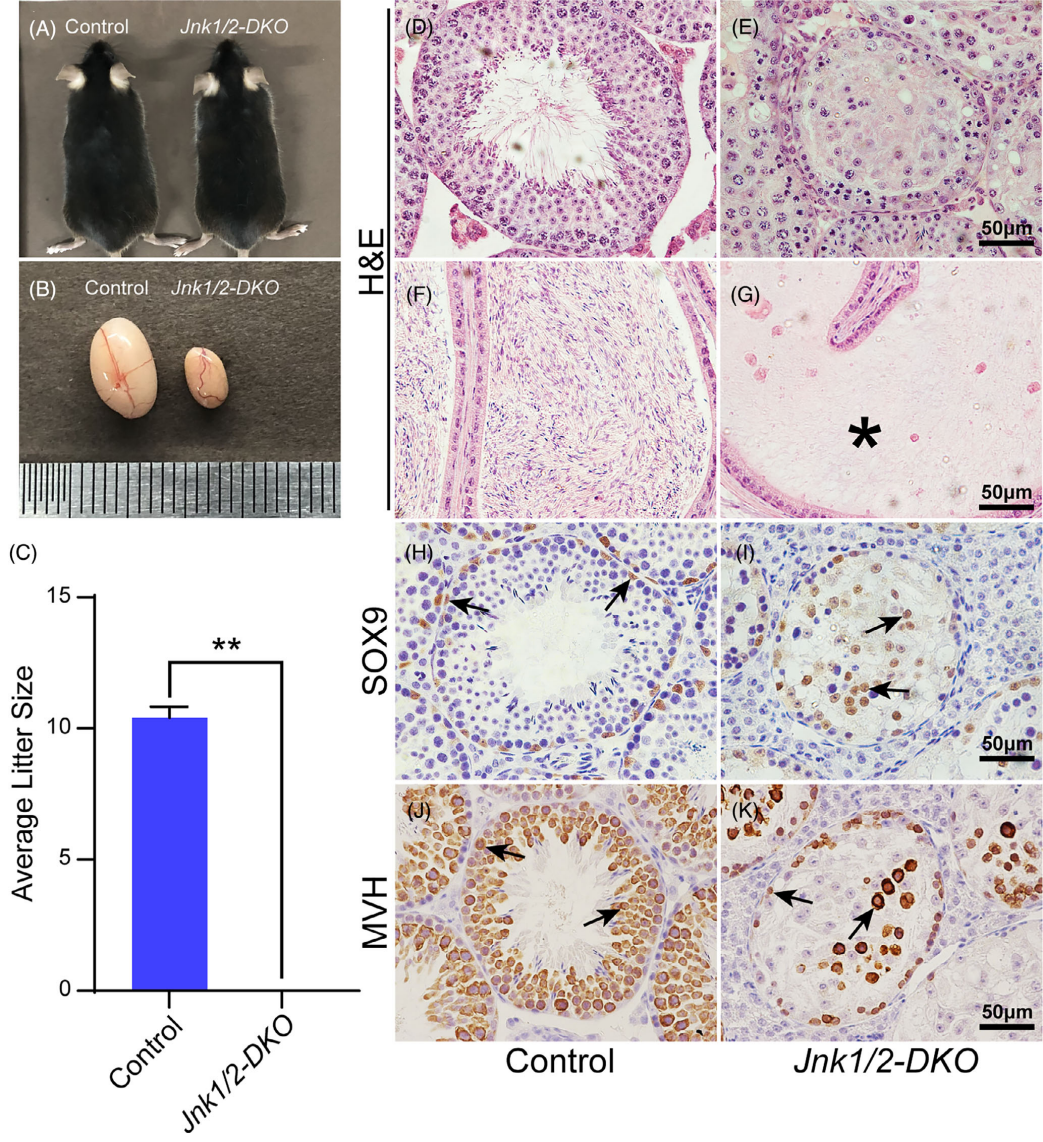
Loss of JNK signalling in Sertoli cells causes male infertility. No developmental abnormalities were observed in *Jnk1/2‐DKO* male mice (A) at 2 months of age, though the testis size was dramatically reduced (B). (C) Fertility test of control and *Jnk1/2‐DKO* male mice. (D–G) H&E staining of testicular and epididymal sections from control and *Jnk1/2‐DKO* mice at 2 months of age. (H,I) The expression of SOX9 (brown, black arrows) in 2‐month‐old control and *Jnk1/2‐DKO* male testes was examined by IHC. (J,K) The expression of MVH (brown, black arrows) in 2‐month‐old control and *Jnk1/2‐DKO* male testes was examined by IHC. The data are presented as the mean ± SEM. *n* = 3; **p < 0.01. Scale bars, 50 μm.

Adult *Jnk1/2‐DKO* mice exhibited grossly normal features (Figure 1A). However, the testes of male *Jnk1/2‐DKO* mice were significantly smaller than control mice (Figure 1B), and the fertility test results showed that *Jnk1/2‐DKO* males were completely infertile (Figure 1C). Histological analysis showed that the cells in the seminiferous tubules of *Jnk1/2‐DKO* mice were disorganised, and no mature sperm were detected in the cauda epididymis (Figure 1D–G). In control testes, SOX9‐positive Sertoli cells were located at the periphery of seminiferous tubules (Figure 1H, black arrows), while in *Jnk1/2‐DKO* mice, these cells were scattered in the centre of seminiferous tubules (Figure 1I, black arrows). The number of MVH‐positive germ cells was drastically reduced, and the remaining germ cells were disorganised in the seminiferous tubules of *Jnk1/2‐DKO* mice (Figure 1J,K).

To investigate the integrity of seminiferous tubules in *Jnk1/2‐DKO* mice, we examined the expression of laminin, a major component of the basal lamina, by immunofluorescence. Both control and *Jnk1/2‐DKO* testes showed an intact laminin‐positive basal lamina (Figure S2A,B). To assess the BTB integrity in *Jnk1/2‐DKO* testes, the biotin tracer was injected into the testicular interstitium. In control testes, the biotin tracer remained in the testicular interstitium, and no signal was noted inside the seminiferous tubules (Figure S2C). By contrast, the biotin tracer signal was detected at the centre of the tubules in *Jnk1/2‐DKO* mice, indicating BTB disruption (Figure S2D). The expression of BTB‐related genes was examined by immunofluorescence and western blot analysis. In control testes, β‐catenin and F‐actin were specifically located at the BTB, whereas these proteins were diffused inside the seminiferous tubule in *Jnk1/2‐DKO* testes (Figure S2E–H). Moreover, the protein levels of N‐cadherin, E‐cadherin, β‐catenin, vimentin, and vinculin were also significantly reduced in *Jnk1/2‐DKO* Sertoli cells (Figure S2I,J).

PLZF‐positive undifferentiated spermatogonia were observed at the periphery of seminiferous tubules in both control and *Jnk1/2‐DKO* mice (Figure S3A,B). STRA8 was detected in spermatocytes at the preleptotene stage in both control and *Jnk1/2‐DKO* mice (Figure S3C,D). The expression of the synaptonemal complex protein SYCP3 was also detected in germ cells from both control and *Jnk1/2‐DKO* mice (Figure S3E,F). Scattered γH2AX signals were detected at the leptotene and zygotene stages and were restricted to the XY body at the pachytene and diplotene stages, with no differences noted between control and *Jnk1/2‐DKO* mice (Figure S3G,H). These results indicated that the initiation of meiosis was not affected in *Jnk1/2‐DKO* mice.

### 3.2 Disorganisation of Sertoli cells in *Jnk1/2‐DKO* mice from P3 onward

**FIGURE 2:**
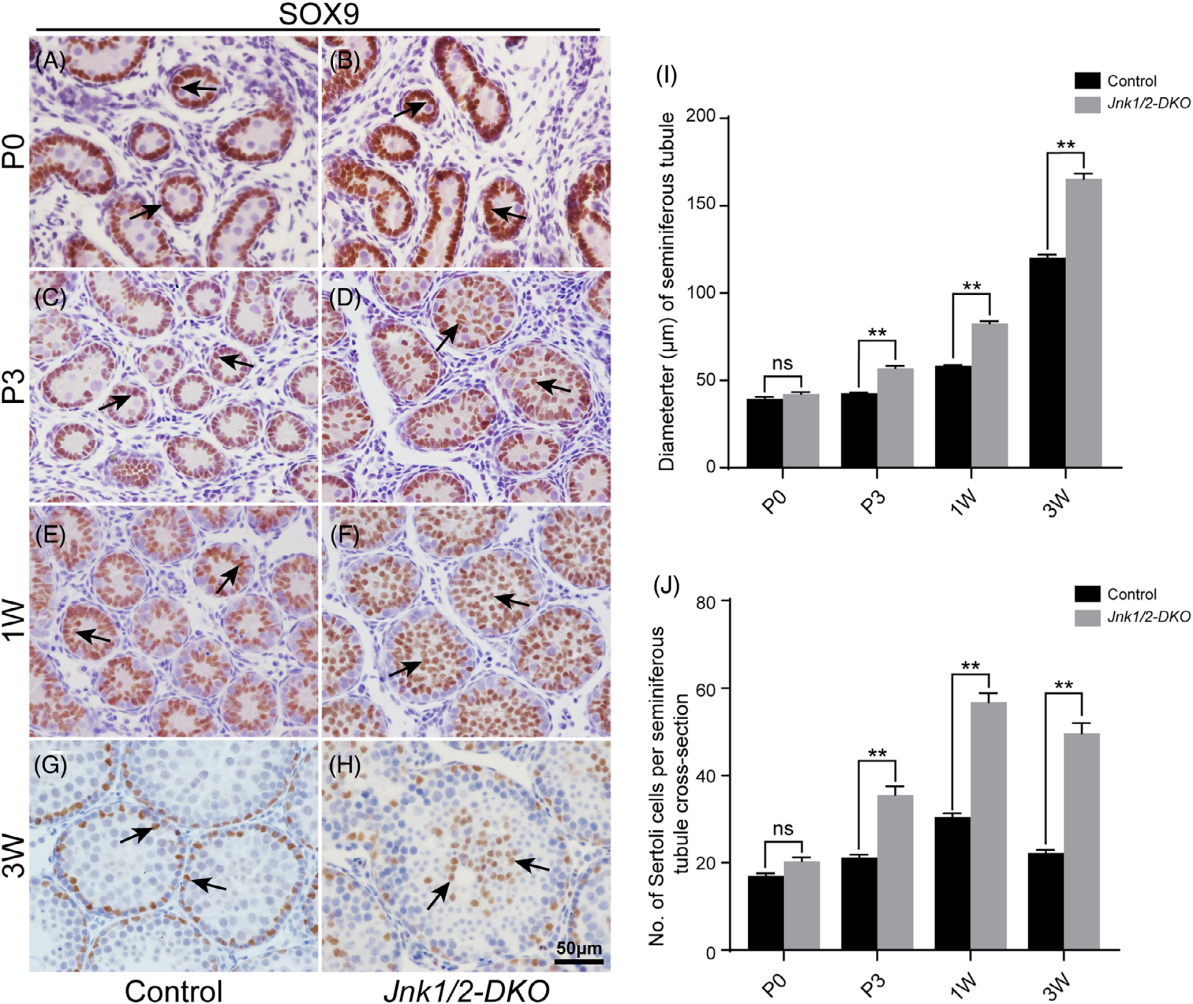
Mislocation of Sertoli cells in *Jnk1/2‐DKO* mice. (A–H) Sertoli cells were labelled with antibody against SOX9 (brown, black arrows) in control and *Jnk1/2‐DKO* testes at P0, P3, 1W, and 3W. (I) Average diameters of seminiferous tubules from control and *Jnk1/2‐DKO* mice at different stages. (J) Average number of SOX9‐positive Sertoli cells per seminiferous tubule cross‐section from control and *Jnk1/2‐DKO* mice at different stages. At least 30 cross‐sections of seminiferous tubules from 6 testicular sections per mouse were counted. The data are presented as the mean ± SEM. *n* = 3; **p < 0.01; ns, not significant. Scale bar, 50 μm.

To explore the underlying mechanisms causing aberrant testis development in *Jnk1/2‐DKO* mice, we examined the location of Sertoli cells and gene expression at different developmental stages. At P0, SOX9‐positive Sertoli cells were located in the peripheral region of seminiferous tubules in both control and *Jnk1/2‐DKO* mice (Figure 2A,B). However, by P3, a large number of Sertoli cell nuclei were found in the centre of seminiferous tubules in *Jnk1/2‐DKO* testes, unlike the peripheral region location in control testes (Figure 2C,D). At 1 week, the lumen of seminiferous tubules in *Jnk1/2‐DKO* mice was filled with Sertoli cell nuclei, unlike the control testes (Figure 2E,F). By 3 weeks, Sertoli cells nuclei were localised along the basement membrane of seminiferous tubules in control mice (Figure 2G), whereas most Sertoli cells still resided in the centre of seminiferous tubules in *Jnk1/2‐DKO* mice (Figure 2H). Statistical analysis showed that the diameter of seminiferous tubules and the number of Sertoli cells significantly increased in *Jnk1/2‐DKO* mice from P3 onward (Figure 2I,J). To assess the proliferation of JNKs‐deficient Sertoli cells, we performed immunostaining for PH3, KI67, and BrdU incorporation. The results in Figure S4 indicate that the number of PH3‐, KI67‐, and BrdU‐positive Sertoli cells was increased in nascent *Jnk1/2‐DKO* testes compared with control testes.

### 3.3 Inactivation of JNK signalling caused senescence of Sertoli cells

**FIGURE 3:**
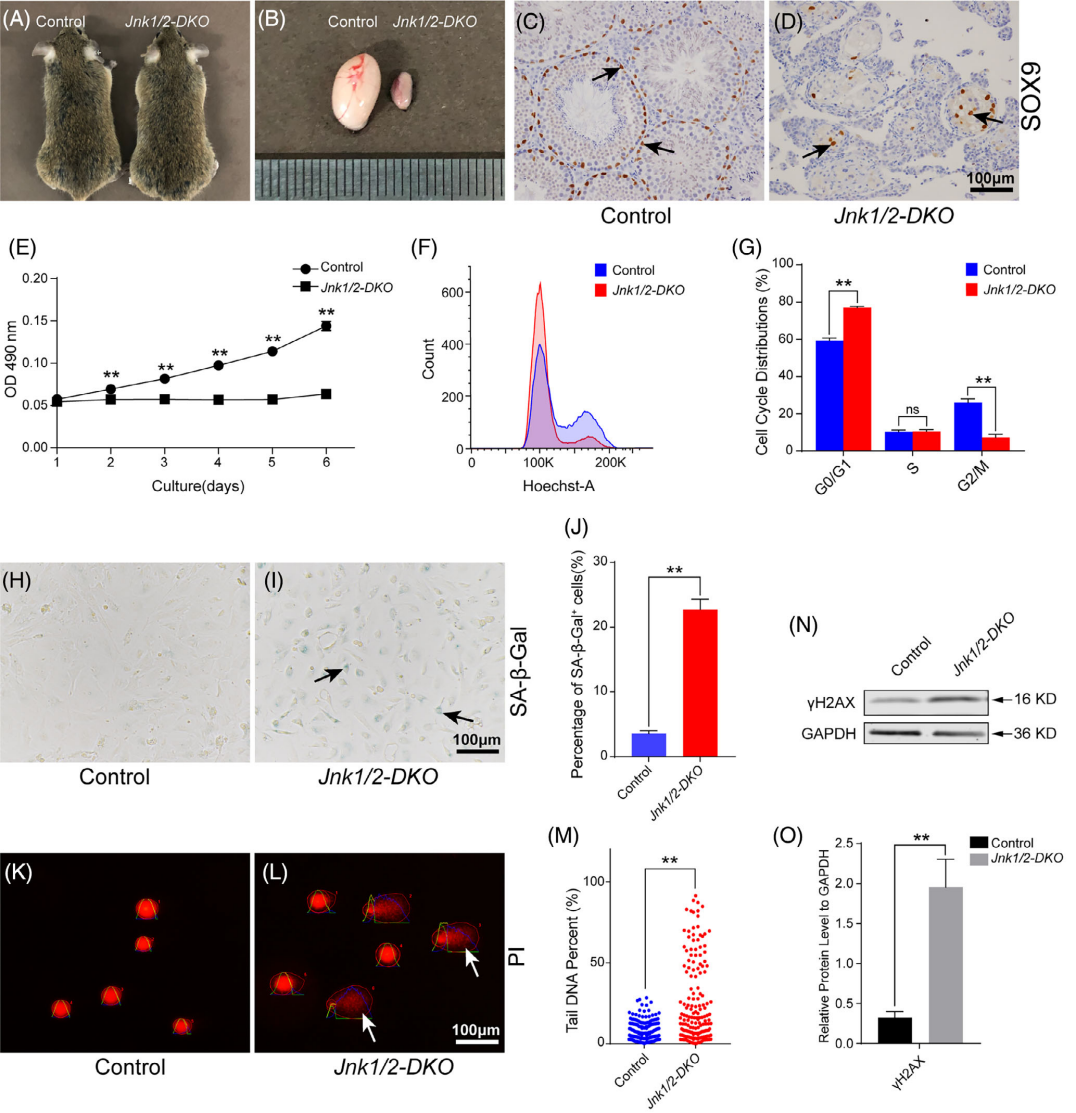
Inactivation of JNK signalling causes senescence in Sertoli cells. (A) No developmental abnormalities were observed in *Jnk1/2‐DKO* male mice at 6 months of age. (B) The size of the testes was dramatically reduced in *Jnk1/2‐DKO* mice at 6 months of age. (C,D) The expression of SOX9 in the testes of 6‐month‐old control and *Jnk1/2‐DKO* male mice was examined by IHC (brown, black arrows). (E) Sertoli cells from control and *Jnk1/2‐DKO* mice were cultured in vitro, and the proliferation was examined by MTT assay. (F,G) Cell cycle analysis of control and *Jnk1/2‐DKO* Sertoli cells was performed via flow cytometry. (H,I) Representative images of SA‐β‐Gal staining in control and *Jnk1/2‐DKO* Sertoli cells (blue, black arrows). (J) Quantitative analysis of SA‐β‐Gal‐positive cells in control and *Jnk1/2‐DKO* Sertoli cells. (K,L) Representative images from the comet assay in control and *Jnk1/2‐DKO* Sertoli cells. Olive tail moments (white arrows) indicate damaged DNA. (M) Quantitative analysis of tail DNA in control and *Jnk1/2‐DKO* Sertoli cells. (N,O) The protein levels of γH2AX in control and *Jnk1/2‐DKO* Sertoli cells were examined by western blotting. The data are presented as the mean ± SEM. *n* = 3; **p < 0.01; ns, not significant. Scale bars, 100 μm.

At 6 months of age, the testis size of *Jnk1/2‐DKO* mice was dramatically reduced (Figure 3A,B), and only a small number of SOX9‐positive Sertoli cells were observed inside the seminiferous tubules (Figure 3C,D). To further explore the functions of JNK signalling in Sertoli cell development, primary Sertoli cells from control and *Jnk1/2‐DKO* mice were cultured in vitro. The purity of cultured primary Sertoli cells was evaluated by IF of SOX9. As shown in Figure S5A–C, the purity of Sertoli cell was 93.4% ± 1.4% (control) and 92.3% ± 1.0% (*Jnk1/2‐DKO*). The results of the MTT assay showed that the proliferation of JNKs‐deficient Sertoli cells was arrested (Figure 3E). Flow cytometry analysis indicated that the loss of JNKs resulted in G0/G1 arrest, accompanied by a concurrent decrease in the G2/M phase (Figure 3F,G). Previous studies have reported that the inactivation of JNK signalling in murine embryonic fibroblasts (MEFs) causes cell senescence.^16^ We examined the activity of senescence‐associated β‐galactosidase (SA‐β‐Gal), a biochemical marker for senescence, in control and *Jnk1/2‐DKO* Sertoli cells.^24^ The percentage of SA‐β‐Gal‐positive Sertoli cells was significantly increased in *Jnk1/2‐DKO* Sertoli cells (22.7% ± 1.6%) compared with control Sertoli cells (3.6% ± 0.4%) (Figure 3H–J). An activated and persistent DNA damage response is another prominent feature of senescence.^24^ The results of the alkaline comet assay showed that olive tail moments in *Jnk1/2‐DKO* Sertoli cells were significantly increased compared with control Sertoli cells (Figure 3K,L). Quantitative analysis also showed that the percentage of tail DNA in *Jnk1/2‐DKO* Sertoli cells was significantly increased compared with control Sertoli cells (Figure 3M). Additionally, the protein level of γH2AX, a phosphorylated DNA damage repair protein, was significantly elevated in *Jnk1/2‐DKO* Sertoli cell (Figure 3N,O).

We also analysed differentially expressed genes using RNA‐Seq. A total of 672 genes were upregulated, and 967 genes were downregulated each with at least twofold changes in *Jnk1/2‐DKO* Sertoli cells compared with control Sertoli cells (Figure S6A). KEGG pathway and GO analyses revealed that the differentially expressed genes were predominantly enriched in cell cycle pathways (Figure S6B,C). The downregulation of cell proliferation‐related genes (e.g., *Ki67*, *Cdk1*, *Cdk6*, *Ccna2*, *Ccnb1*, *Ccnb2*, *Ccnd1*, *Ccnd2*, and *Ccnd3*) in *Jnk1/2‐DKO* Sertoli cells was further verified by qRT‐PCR (Figure S6D,E). Collectively, these results indicate that the inactivation of JNK signalling causes Sertoli cell senescence.

### 3.4 The defect of Sertoli cells development in *Jnk1/2‐DKO* mice was partially mediated by *c‐Jun*

**FIGURE 4:**
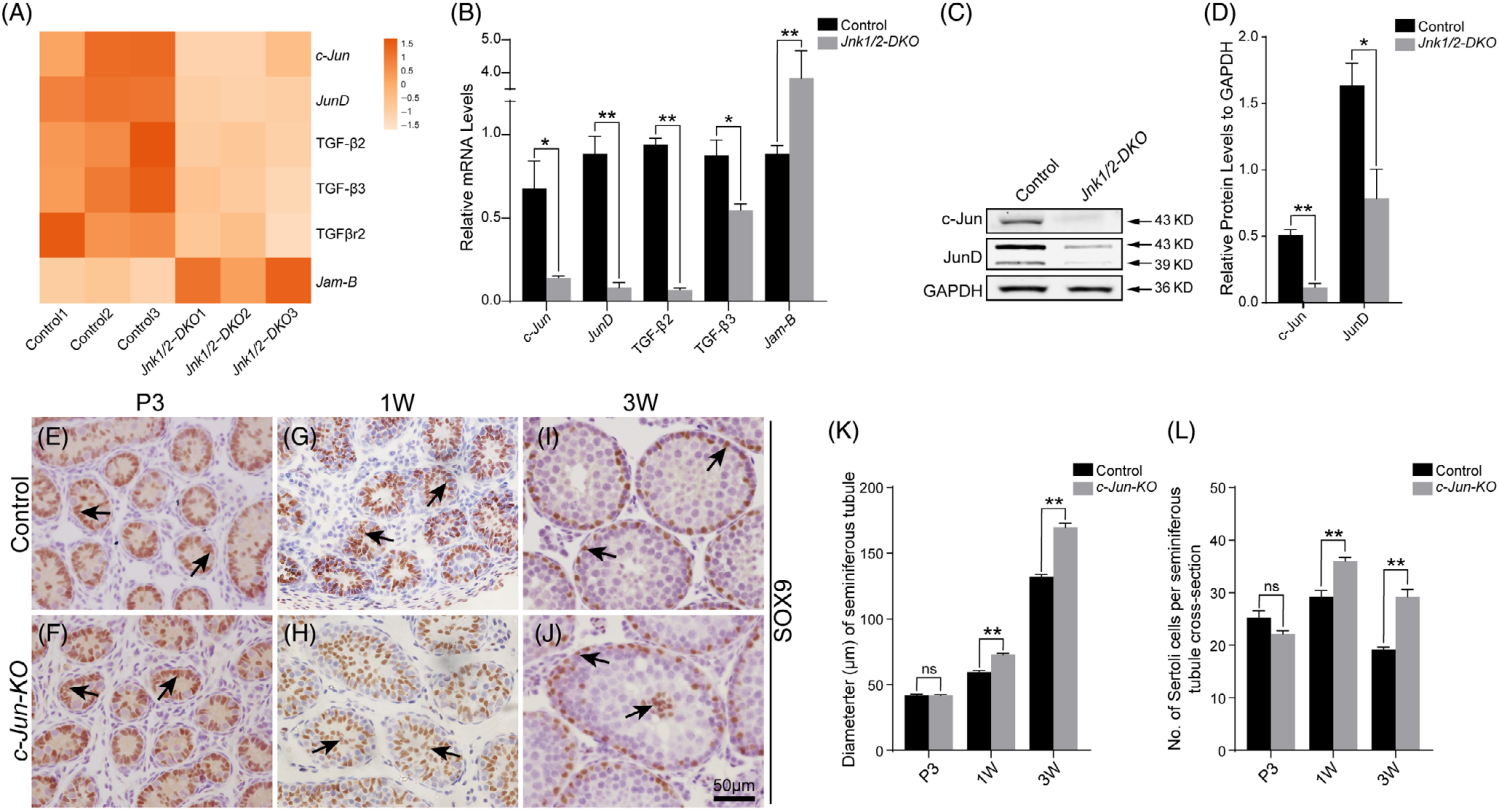
Mislocation of Sertoli cells is observed in *c‐Jun‐KO* testes. (A) Heatmap of RNA‐Seq data from control and *Jnk1/2‐DKO* Sertoli cells. (B) The transcriptional levels of *c‐Jun*, *JunD*, TGF‐β2, TGF‐β3, and *Jam‐B* in control and *Jnk1/2‐DKO* Sertoli cells were examined by qRT‐PCR. (C,D) The protein levels of c‐Jun and JunD in control and *Jnk1/2‐DKO* Sertoli cells were examined by western blotting. (E–J) Sertoli cells were labelled with antibody against SOX9 (brown, black arrows) in control and *c‐Jun‐KO* testes at P3, 1W, and 3W. (K) Average diameters of seminiferous tubules from control and *c‐Jun‐KO* mice at various stages. (L) Average number of SOX9‐positive Sertoli cells per seminiferous tubule cross‐section from control and *c‐Jun‐KO* mice at various stages. At least 30 cross‐sections of seminiferous tubules from 6 testicular sections per mouse were counted. The data are presented as the mean ± SEM. *n* = 3; *p < 0.05; **p < 0.01; ns, not significant. Scale bar, 50 μm.

RNA‐seq analysis showed that *c‐Jun* and *JunD* were downregulated in JNKs‐deficient Sertoli cells (Figure 4A). These results were further verified by qRT‐PCR (Figure 4B) and western blot analysis (Figure 4C,D). *c‐Jun* and *JunD* are canonical substrates of JNK, and their inactivation also causes senescence in MEFs.^25^, ^26^, ^27^, ^28^, ^29^ However, Sertoli cell development is not affected in *JunD* ^*Δ/Δ*^ mice.^30^ To test whether the defects of Sertoli cell development in *Jnk1/2‐DKO* mice were caused by the downregulation of *c‐Jun* expression, we specifically deleted *c‐Jun* in Sertoli cells using *c‐Jun* ^*flox/flox*^ *;Amh* ^*+/Cre*^ (*c‐Jun‐KO*) mice. At P3, Sertoli cell nuclei were located at the periphery of the seminiferous tubules in both control and *c‐Jun‐KO* mice (Figure 4E,F). Mislocation of Sertoli cells was observed in *c‐Jun‐KO* testes at 1 and 3 weeks, with a large number of Sertoli cells found at the centre of the seminiferous tubules, compared with control mice, where they remained at the periphery (Figure 4G–J). Statistical analysis showed a substantial increase in both seminiferous tubule diameter and Sertoli cell number in *c‐Jun‐KO* mice (Figure 4K,L), consistent with the findings in *Jnk1/2‐DKO* mice. However, massive Sertoli cell loss was not observed in adult *c‐Jun‐KO* mice (Figure S7). To further explore the functions of *c‐Jun* in Sertoli cell development, primary Sertoli cells from control (92.6% ± 0.2%) and *c‐Jun‐KO* mice (91.8% ± 1.5%) were cultured in vitro (Figure S5D–F). DNA damage was also not detected by the comet assay (Figure S8A–C), and the protein level of γH2AX was not increased in *c‐Jun‐KO* Sertoli cells (Figure S8D,E). No significant decrease in proliferation or cell cycle arrest was detected in *c‐Jun‐KO* Sertoli cells by MTT assay and FACS analysis (Figure S8F–J). These results suggest that inactivation of *c‐Jun* caused defects in the establishment of polarity in Sertoli cells, but not cell senescence.

### 3.5 *c‐Jun* was involved in polarity establishment in Sertoli cells by regulating the expression of TGF‐β2 and *Jam‐B*

**FIGURE 5:**
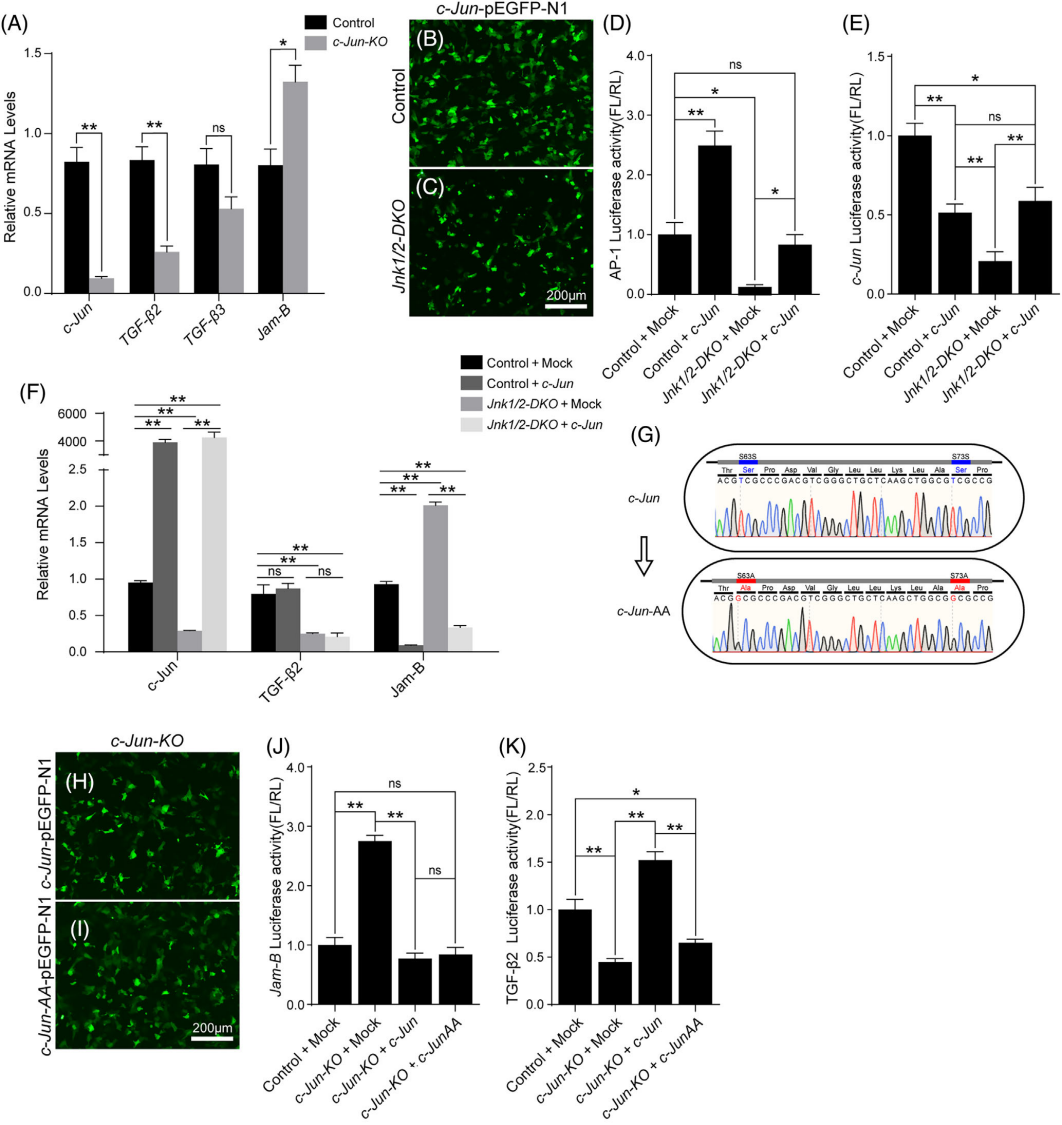
*c‐Jun* is involved in the establishment of polarity in Sertoli cell by regulating the expression of TGF‐β2 and *Jam‐*B. (A) The transcriptional levels of *c‐Jun*, TGF‐β2, TGF‐β3, and *Jam‐B* in control and *c‐Jun‐KO* Sertoli cells were examined by qRT‐PCR. Sertoli cells isolated from control (B) and *Jnk1/2‐DKO* (C) mice were cultured and transfected with *c‐Jun* overexpression vector. Luciferase activity analysis of AP‐1 (D) and *c‐Jun* promoter (E) in control and *Jnk1/2‐DKO* Sertoli cells with or without *c‐Jun* overexpression. (F) The transcriptional levels of *c‐Jun*, TGF‐β2, and *Jam‐B* in control and *JNK1/2‐DKO* Sertoli cells with or without *c‐Jun* overexpression was examined by qRT‐PCR. (G) A schematic diagram of the mutation at the phosphorylation site in the *c‐Jun* protein. Sertoli cells isolated from *c‐Jun‐KO* mice were cultured and transfected with the *c‐Jun* (H) or *c‐Jun‐AA* overexpression vector (I). Luciferase activity analysis of the *Jam‐B* (J) and TGF‐β2 promoter (K) in control and *c‐Jun‐KO* Sertoli cell with *c‐Jun* or *c‐Jun‐AA* overexpression. The data are presented as the mean ± SEM. *n* = 3; *p < 0.05; **p < 0.01; ns, not significant. Scale bars, 200 μm.

RNA‐seq analysis revealed that the loss of JNK signalling in Sertoli cells leads to the downregulation of the TGF‐β signalling pathway (TGF‐β2, TGF‐β3, and TGF‐β receptor 2) and a significant increase in *Jam‐B* (Figures 4A and S6B). TGF‐β signalling is crucial for maintaining cell polarity, as observed in neurons, dermal papilla fibroblasts, and mammary gland epithelial cells.^31^, ^32^, ^33^ *Jam‐B* is also involved in establishing cell polarity and forming tight junctions by recruiting ZO‐1 and PAR3.^34^, ^35^, ^36^, ^37^ qRT‐PCR analysis showed that TGF‐β2 expression was significantly decreased, and *Jam‐B* expression was increased in *c‐Jun‐KO* Sertoli cells (Figure 5A), consistent with the findings in *Jnk1/2‐DKO* Sertoli cells (Figure 4A,B).

It is well established that JNKs regulate gene expression by phosphorylating and activating AP‐1 and AP‐1‐related transcription factors (e.g., *c‐Jun*, *JunB*, *JunD*, and ATF2).^15^, ^16^ Therefore, it is likely that JNKs regulate the transcriptional activity of TGF‐β2 and *Jam*‐B through *c‐Jun*/AP‐1. Luciferase assay showed a significant decrease in AP‐1 activity in *Jnk1/2‐DKO* Sertoli cells, which could be rescued by *c‐Jun* overexpression (Figure 5B–D). Similarly, there was a notable reduction in *c‐Jun* promoter activity in *Jnk1/2‐DKO* Sertoli cells. *c‐Jun* overexpression significantly enhanced *c‐Jun* promoter activity in *Jnk1/2‐DKO* Sertoli cells (Figure 5E). However, *c‐Jun* overexpression in *Jnk1/2‐DKO* Sertoli cells led to a significant decrease in *Jam‐B* mRNA but not TGF‐β2 mRNA (Figure 5F). We postulate that the modulation of TGF‐β2 transcription by *c‐Jun*/AP‐1 might depend on JNKs phosphorylation. To test whether *c‐Jun* regulation of TGF‐β2 expression is dependent on JNKs phosphorylation, we constructed *c‐Jun* overexpression vector carrying two mutated serine phosphorylation sites (Ser63 and Ser73) of JNKs, termed *c‐Jun‐AA* (Figure 5G). The luciferase assay results showed that *Jam‐B* promoter activity was repressed by both *c‐Jun* and *c‐Jun‐AA* overexpression in *c‐Jun‐KO* Sertoli cells, indicating that *c‐Jun* regulates *Jam‐B* expression independently of JNKs phosphorylation (Figure 5H–J). However, TGF‐β2 promoter activity was significantly induced by *c‐Jun* but not by *c‐Jun‐AA* in *c‐Jun‐KO* Sertoli cells (Figure 5K), indicating that *c‐Jun*‐induced TGF‐β2 expression in Sertoli cells is dependent on JNKs phosphorylation.

### 3.6 Inactivation of JNKs caused P27^KIP1^ ‐dependent cell senescence in Sertoli cells

**FIGURE 6:**
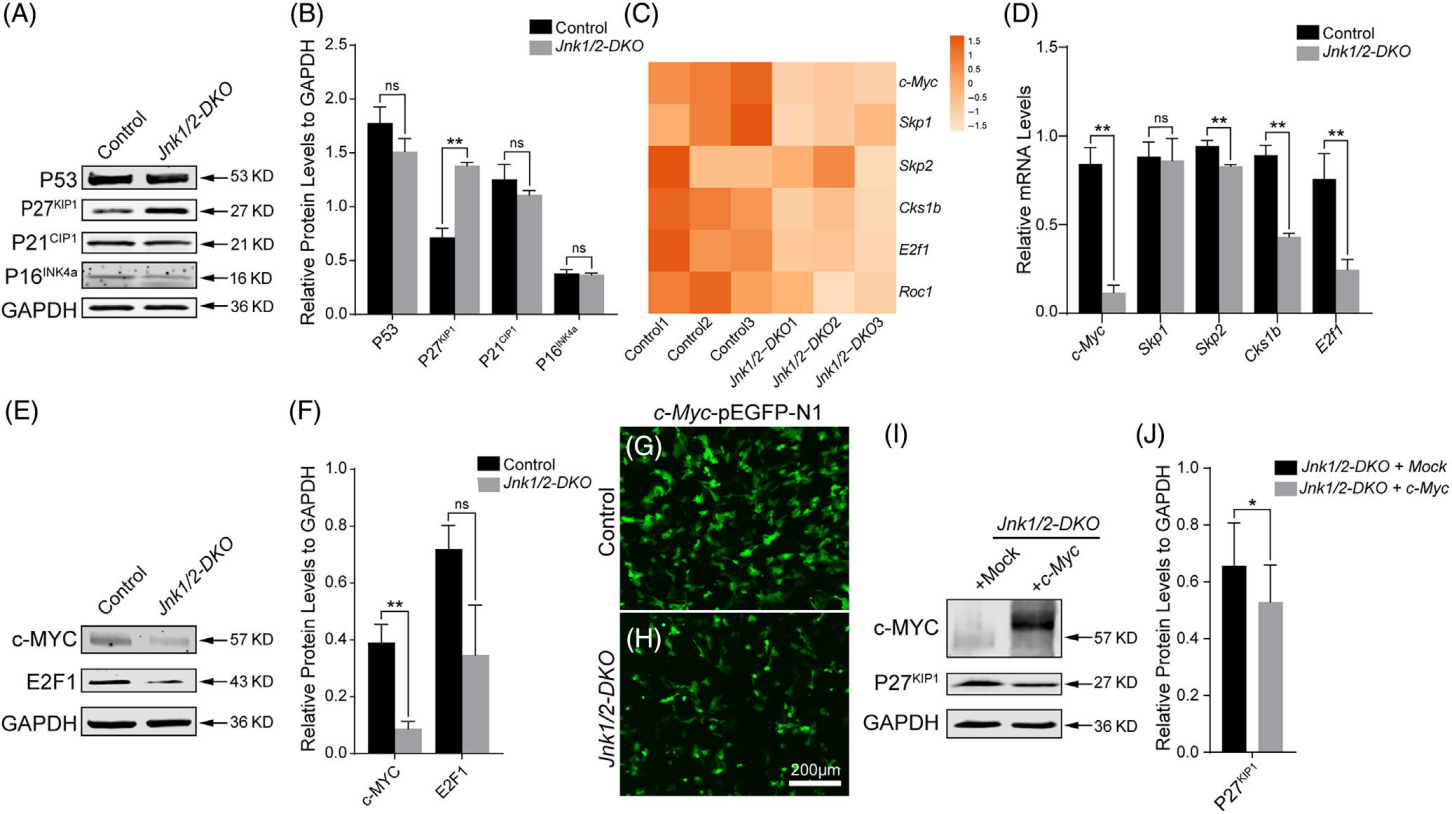
The proteolysis of P27^KIP1^ in Sertoli cells is regulated by JNK signalling pathway. (A,B) The protein levels of P53, P27^KIP1^, P21^CIP1^, and P16^INK4a^ were examined by western blotting. (C) Heatmap of RNA‐Seq data from control and *Jnk1/2‐DKO* Sertoli cells. (D) The transcriptional levels of *c‐Myc*, *Skp1*, *Skp2*, *Cks1b*, and *E2f1* in control and *JNK1/2‐DKO* Sertoli cells were examined by qRT‐PCR. (E,F) The protein levels of c‐MYC and E2F1 in control and *Jnk1/2‐DKO* Sertoli cells were examined by western blotting. Sertoli cells isolated from control (G) and *Jnk1/2‐DKO* (H) mice were cultured and transfected with *c‐Myc* overexpression vector. (I,J) The protein level of P27^KIP1^ in *Jnk1/2‐DKO* Sertoli cells with or without *c‐Myc* overexpression was examined by western blotting. The data are presented as mean ± SEM. *n* = 3; *p < 0.05; **p < 0.01; ns, not significant. Scale bar, 200 μm.

To elucidate the underlying mechanism of cell senescence observed in *Jnk1/2‐DKO* Sertoli cells, we examined the expression of genes known to be involved in cell cycle control and cell senescence. It has been reported that P53, P21^CIP1^, P16^INK4a^, and P27^KIP1^ are regarded as key effectors of cellular senescence.^38^, ^39^, ^40^ We found that the protein levels of P53, P21^CIP1^, and P16^INK4a^ did not change in *Jnk1/2‐DKO* Sertoli cells compared with the control Sertoli cells. However, the protein level of P27^KIP1^ significantly increased in *Jnk1/2‐DKO* Sertoli cells (Figure 6A,B). Previous studies have reported that *c‐Myc*, a classical substrate of JNKs, facilitates the proteolysis of P27^KIP1^ protein through the SKP2 ubiquitin‐proteasome complex with involvement of *E2f1*.^41^, ^42^, ^43^, ^44^, ^45^ The results of RNA‐Seq analysis showed that *c‐Myc*, *E2f1*, and *Cks1b* (a component of SKP2 ubiquitin‐proteasome complex) were significantly decreased in *Jnk1/2‐DKO* Sertoli cells (Figure 6C), and these results were further validated by qRT‐PCR (Figure 6D) and western blot analysis (Figure 6E,F). To test whether the protein level of P27^KIP1^ could be repressed by *c‐Myc*, *c‐Myc* was overexpressed in *Jnk1/2‐DKO* Sertoli cells. We found that overexpression of *c‐Myc* in *Jnk1/2‐DKO* Sertoli cells significantly repressed the protein level of P27^KIP1^ (Figure 6G–J). These results suggest that inactivation of JNK signalling causes senescence in Sertoli cells, most likely due to the aberrant proteolysis of P27^KIP1^ mediated by *c‐Myc*.

## 4 DISCUSSION AND CONCLUSIONS

The JNK signalling cascade is an evolutionarily conserved pathway, widely recognised for its pivotal role in regulating cell proliferation, migration, and apoptosis.^9^, ^12^ Previous studies suggest a likely involvement of JNK signalling in spermatogenesis.^17^, ^18^, ^19^, ^20^ However, the exact function of JNK signalling in male reproduction remains unclear. Our study reveals critical roles of JNK signalling in Sertoli cell development. Disruption of JNK signalling leads to loss of polarity and cell senescence in Sertoli cells, resulting in aberrant testis development and male infertility in mouse models. Our study also suggests that disturbance of the JNK signalling pathway is probably one of the etiological causes of male infertility in human patients.

*c‐Jun*, a canonical substrate of JNK, showed significantly reduced expression in *Jnk1/2‐DKO* Sertoli cells. Double knockout of *Jnk1* and *Jnk2* caused loss of polarity and senescence in Sertoli cells. However, only polarity loss was detected in *c‐Jun‐*deficient Sertoli cells, unlike in MEFs where inactivation of *c‐Jun* also increases cell senescence.^25^, ^26^, ^27^, ^28^, ^29^ Our findings suggest that JNK signalling plays different roles in Sertoli cell development, with *c‐Jun* most likely mediating polarity establishment.

Sertoli cells are the most important somatic cells in the testis, providing nutritional and physical support for spermatogenesis. They are highly polarised epithelial cells, and the establishment and maintenance of this polarity are essential for their function. It has been reported that *Wt1* is involved in polarity establishment in Sertoli cells by modulating Wnt‐PCP signalling, and deletion of *Wt1* causes loss of polarity in Sertoli cells, leading to germ cell loss and male infertility.^46^ Rho GTPase signalling also participates in Sertoli cell polarity establishment, deletion of *Rac1* or *Cdc42* in Sertoli cells causes severe cell polarity defects and male infertility.^47^, ^48^ Additionally, altered LKB1/AMPK/TSC1/TSC2/mTOR signalling disrupts Sertoli cell polarity and spermatogenesis.^49^, ^50^

TGF‐β signalling also plays a vital role in maintaining polarity in various cell types. It initiates neuronal polarity by directing naïve neurites into axons through site‐specific phosphorylation of PAR6.^31^ Additionally, TGF‐β signalling is essential for maintaining polarity in dermal papilla fibroblasts and mammary gland epithelial cells.^32^, ^33^ In this study, we found a downregulation of TGF‐β2 in both *Jnk1/2‐DKO* and *c‐Jun*‐*KO* Sertoli cells. Our study also demonstrated that *c‐Jun* induced TGF‐β2 expression in Sertoli cells is dependent on JNKs phosphorylation. Studies have shown that both TGF‐β2 and TGF‐β3 inhibit the expression of *Jam‐B*, which is involved in cell polarity establishment and tight junction formation.^51^, ^52^ In endothelial cells, JAM‐B is localised at cell junctions and participates in the establishment of cell polarity and the formation of tight junctions by recruiting PAR3 and ZO‐1.^34^, ^35^ In Sertoli cells, Jam‐B is involved in recruiting polarity proteins and interacts with JAM‐C, potentially contributing to cell polarity establishment and spermatogenesis.^36^, ^37^ In this study, we observed upregulation of *Jam‐B* in both *Jnk1/2‐DKO* and *c‐Jun*‐*KO* Sertoli cells. We speculated that the increased JAM‐B may also impede the establishment of Sertoli cell polarity. However, further investigation is needed to verify this hypothesis. Based on these results, we concluded that the loss of cell polarity in JNKs‐deficient Sertoli cells is likely due to the downregulation of TGF‐β2, and the upregulation of *Jam‐B* may also be involved in this process.

Cellular senescence, induced by various stresses, leads to stable cell cycle arrest, decreased tissue regeneration and inflammation, and is associated with diabetes, neurodegenerative diseases, and tumorigenesis. Sertoli cells are susceptible to age‐associated dysfunction in the male reproductive system.^53^ Aging Sertoli cells exhibit reduced quantity and metabolic disturbances, potentially contributing to spermatogenic failure and male infertility.^54^ However, the underlying mechanisms regulating Sertoli cell aging remain unclear. Increasing studies suggest that the JNK signalling pathway plays a significant role in cellular senescence. Activation of JNK signalling promotes osteoblast senescence by repressing collagen synthesis.^55^ Conversely, inactivation of JNK signalling causes senescence in MEFs, mediated by P53.^56^ Deletion of *Jnk1* and *Jnk2* in joint tissues increases the expression of senescence marker P16^INK4a^ and enhances senescence.^57^ In this study, we found that the expression of P16^INK4a^, P21^Cip1^, and P53 did not change in *Jnk1/2‐DKO* Sertoli cells, whereas the protein level of P27^KIP1^ was significantly increased. Additionally, we observed a reduction in the expression of *c‐Myc* in JNKs‐deficient Sertoli cells. Previous reports have highlighted that c‐MYC mediates the proteolysis of P27^KIP1^ through the ubiquitin‐proteasome complex.^41^, ^42^, ^43^, ^44^, ^45^ Our study also demonstrated that overexpression of *c‐Myc* in JNKs‐deficient Sertoli cells reduced the protein level of P27^KIP1^. Based on these results, we concluded that cell senescence in JNKs‐deficient Sertoli cells is probably caused by increased P27^KIP1^, and the upregulation of P27^KIP1^ is most likely due to the downregulation of *c‐Myc* gene.

**FIGURE 7:**
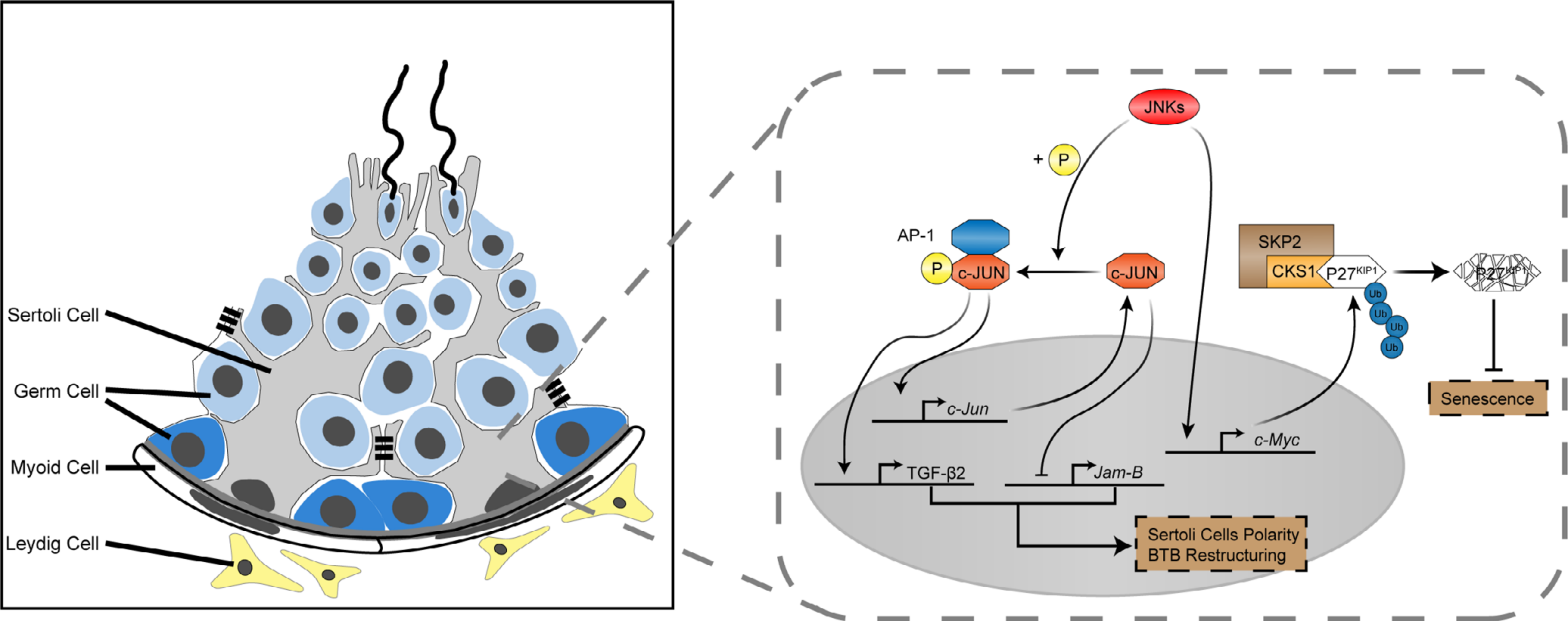
Schematic diagram of JNK signalling in Sertoli cell development. JNK signalling plays dual roles in Sertoli cell development. JNKs are involved in polarity establishment by regulating TGF‐β2 and *Jam‐B* expression which is mediated by *c‐Jun*/AP‐1. The senescence of JNKs‐deficient Sertoli cells is caused by c‐MYC‐mediated proteolysis of P27^KIP1^.

In summary, our study demonstrates that JNK signalling plays a critical role in the development and functional maintenance of Sertoli cells. Inactivation of JNK signalling leads to polarity loss and cell senescence in Sertoli cells. Mechanistic studies revealed that JNK signalling is probably involved in the establishment of Sertoli cell polarity by regulating the expression of TGF‐β2 and *Jam‐B*, which is dependent on *c‐Jun*. Cell senescence in JNKs‐deficient Sertoli cells is likely caused by increased P27^KIP1^, and the upregulation of P27^KIP1^ is most likely due to the downregulation of the *c‐Myc* gene (Figure 7). Our findings provide insights into the regulation of Sertoli cell development and functional maintenance.

## AUTHOR CONTRIBUTIONS

*Conceptualisation*: Fei Gao, Yuhua Shi, and Jian Hou. *Methodology*: Min Chen and Xiuhong Cui. *Investigation*: Zhiming Shen, Yang Gao, Yang Gao, Nan Wang, Changhuo Cen, Mengyue Wang, Bowen Liu, and Jiayi Li. *Visualisation*: Zhiming Shen, Yang Gao, and Yang Gao. *Writing—original draft*: Zhiming Shen and Yang Gao. *Writing—review and editing*: Fei Gao, Yuhua Shi, and Jian Hou. *Funding acquisition*: Fei Gao. *Supervision*: Fei Gao, Yuhua Shi, and Jian Hou.

## FUNDING INFORMATION

This study was supported by the Strategic Priority Research Program of the Chinese Academy of Sciences (XDB0840000), the National Natural Science Foundation of China (82421003, 32270902, 32170855, and 32300678), the Faculty Resources Project of the College of Life Sciences, Inner Mongolia University (2022‐104), and the China Postdoctoral Science Foundation (2023M730748).

## CONFLICT OF INTEREST STATEMENT

The authors declare that they have no conflict of interest.

## Supporting information


**Figure S1.** JNK signalling is inactivated in Sertoli cells of *Jnk1/2‐DKO* mice.
**Figure S2.** BTB structure is disrupted in *Jnk1/2‐DKO* testis.
**Figure S3.** The process of meiosis is not affected in *Jnk1/2‐DKO* mice.
**Figure S4.** The proliferation of Sertoli cells is increased in nascent *Jnk1/2‐DKO* mice.
**Figure S5.** Purity of cultured primary Sertoli cells.
**Figure S6.** Differentially expressed genes in Sertoli cells of *Jnk1/2‐DKO* mice.
**Figure S7.** No defects of germ cell development are observed in adult *c‐Jun‐KO* males.
**Figure S8.** Inactivation of *c‐Jun* does not cause senescence in Sertoli cells.
**Table S1.** List of antibodies used in this study.
**Table S2.** List of primers used for plasmid construction.
**Table S3.** List of primers used for qRT‐PCR.

## Data Availability

The data that support the findings of this study are available from the corresponding author upon reasonable request.
