# Supplementary material for: Inactivation of JNK signalling results in polarity loss and cell senescence of Sertoli cell
Source: Cell Prolif. 2024 Sep 27;58(2):e13760. doi: 10.1111/cpr.13760 (PMC11839192; doi:10.1111/cpr.13760)
Supplement: Supplementary file 1 — Figure S1. JNK signalling is inactivated in Sertoli cells of Jnk1/2‐DKO mice. Figure S2. BTB structure is disrupted in Jnk1/2‐DKO testis. Figure S3. The process of meiosis is not affected in Jnk1/2‐DKO mice. Figure S4. The proliferation of Sertoli cells is increased in nascent Jnk1/2‐DKO mice. Figure S5. Purity of cultured primary Sertoli cells. Figure S6. Differentially expressed genes in Sertoli cells of Jnk1/2‐DKO mice. Figure S7. No defects of germ cell development are observed in adult c‐Jun‐KO males. Figure S8. Inactivation of c‐Jun does not cause senescence in Sertoli cells. Table S1. List of antibodies used in this study. Table S2. List of primers used for plasmid construction. Table S3. List of primers used for qRT‐PCR. [file CPR-58-e13760-s001.docx]

Supplementary Information for

**Inactivation of JNK signaling results in polarity loss and cell senescence of Sertoli cell**

Zhiming Shen, Yang Gao, Xuedong Sun, Min Chen, Changhuo Cen, Mengyue Wang, Nan Wang, Bowen Liu, Jiayi Li, Xiuhong Cui, Jian Hou^*^, Yuhua Shi^*^, Fei Gao^*^

^*^Correspondence: [gaof@ioz.ac.cn](mailto:gaof@ioz.ac.cn) (F.G.); [shiyuhua@gdph.org.cn](mailto:shiyuhua@gdph.org.cn) (Y.S.); [houjian@cau.edu.cn](mailto:houjian@cau.edu.cn) (J.H.)

This PDF file includes:

Fig. S1-S8

Table S1-S3

##
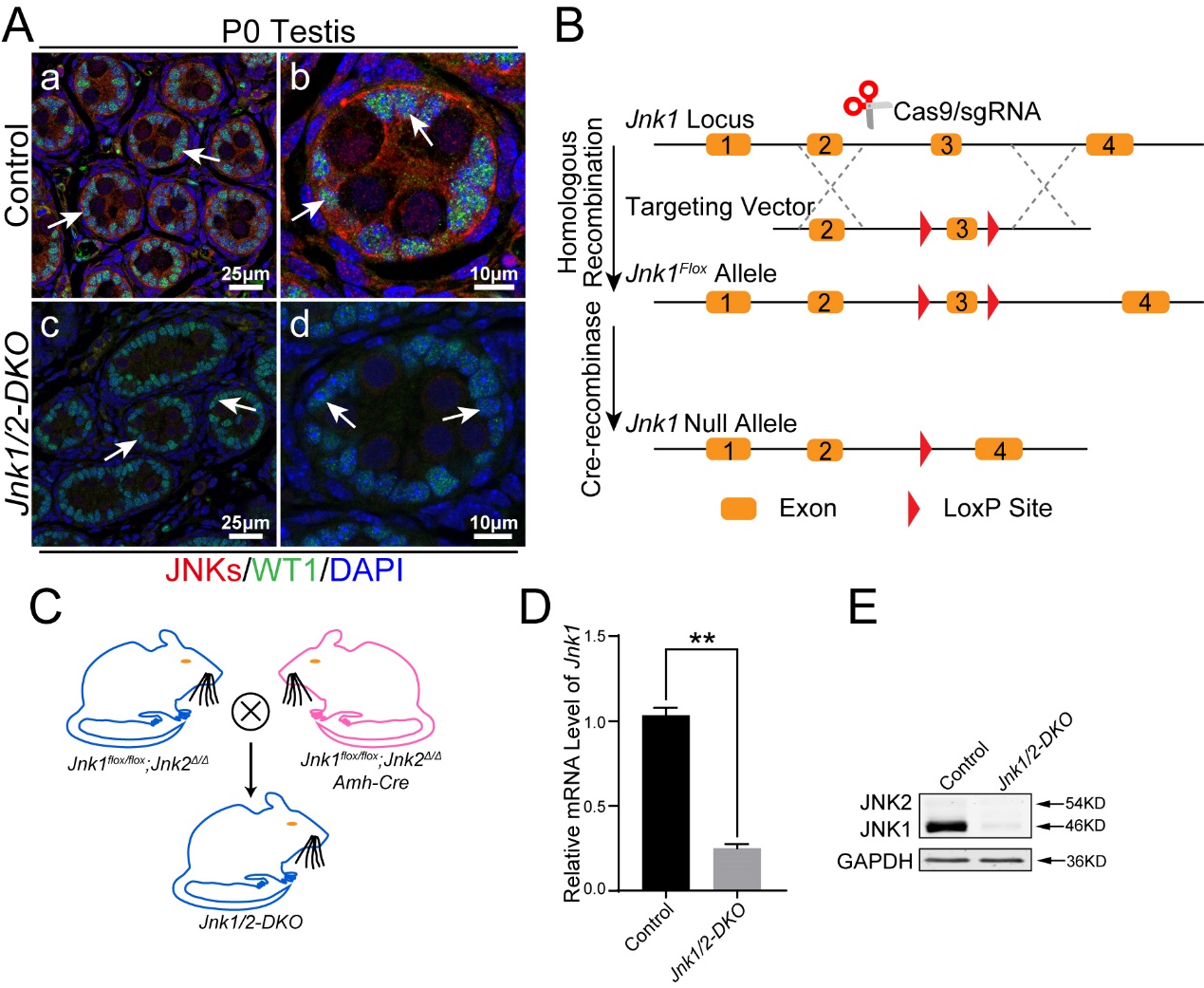
Fig. S1 JNK signaling is inactivated in Sertoli cells of *Jnk1/2-DKO* mice.

(**A**) The expression of JNKs (red) and WT1 (green, white arrows) in the testes of control and *Jnk1/2-DKO* mice was examined by immunofluorescence (IF) at P0. Scale bars: 25 μm and 10 μm (zoomed-in image) (**B**) Schematic diagram of the strategy for generating the *Jnk1^flox^* mouse strain. (**C**) *Jnk1^flox/flox^;Jnk2^Δ/Δ^* males mice were crossed with *Jnk1^flox/flox^;Jnk2^Δ/Δ^;Amh-Cre* female mice to obtain *Jnk1/2-DKO* male mice. (**D**) The expression of *Jnk1* in P7 Sertoli cells was examined by qRT-PCR. (**E**) The protein levels of JNKs in P7 *Jnk1/2-DKO* Sertoli cells was examined by western blotting. Since *Jnk1^flox/flox^;Jnk2^Δ/Δ^* male mice were used as control, only a 45kDa band of JNK1 could be detected. The data are presented as the mean ± SEM. n = 3; **p < 0.01.

##
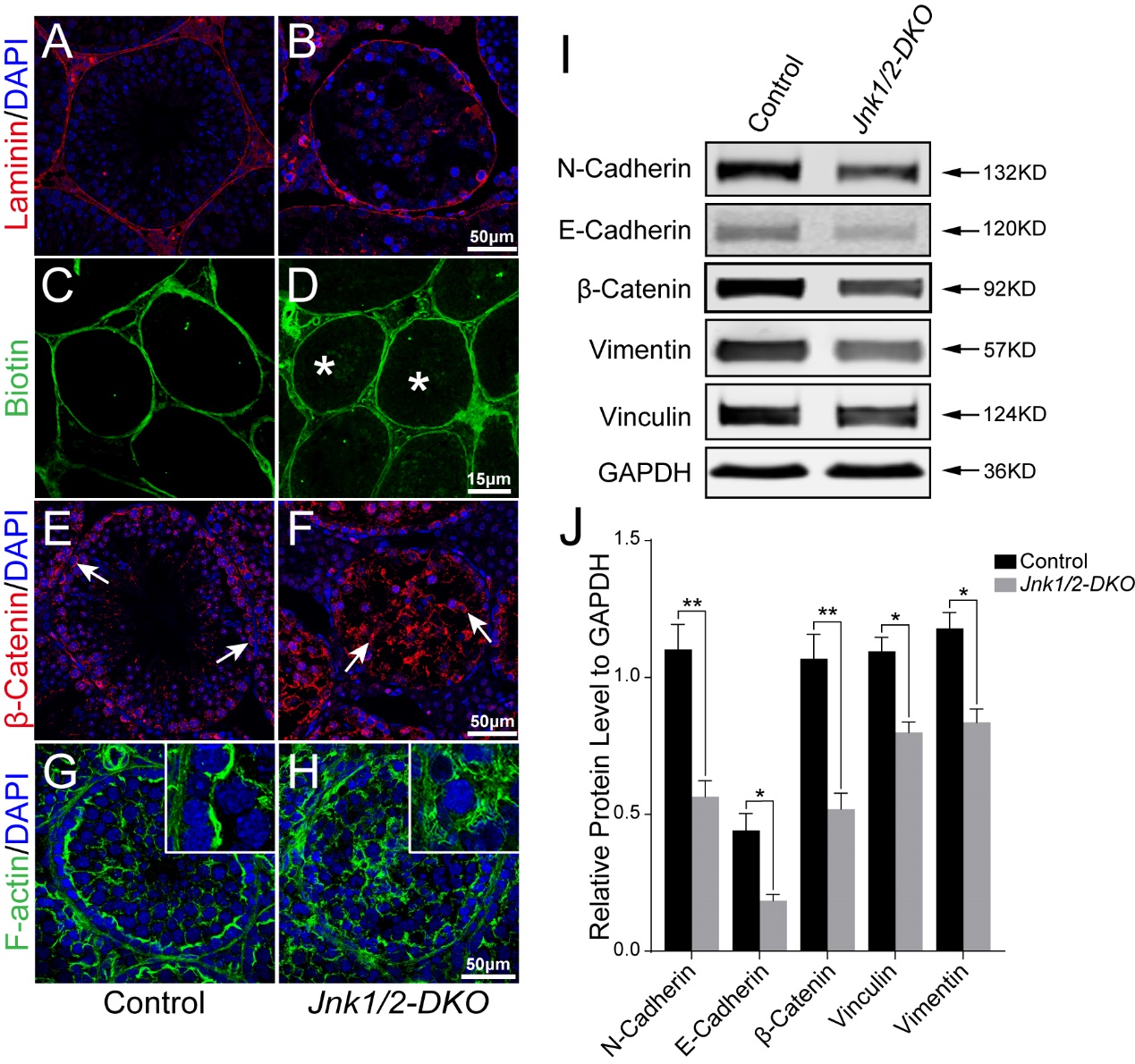
Fig. S2. BTB structure is disrupted in *Jnk1/2-DKO* testis.

The basal membrane of the seminiferous tubules was labeled with Laminin in both control (**A**) and *Jnk1/2-DKO* testes (**B**). Four-weeks-old control (**C**) and *Jnk1/2-DKO* (**D**) testes were injected with biotin and detected with streptavidin. Scale bar: 15 μm. The expression of β-Catenin (red, white arrows) in control (**E**) and *Jnk1/2-DKO* testes (**F**) was examined by IF. F-actin (green) in control (**G**) and *Jnk1/2-DKO* testes (**H**) was labeled with phalloidin. (**I**, **J**) The protein levels of BTB assembly-related proteins were analyzed by western blot. The data were presented as mean ± SEM. n=3; *p < 0.05; **p < 0.01.

##
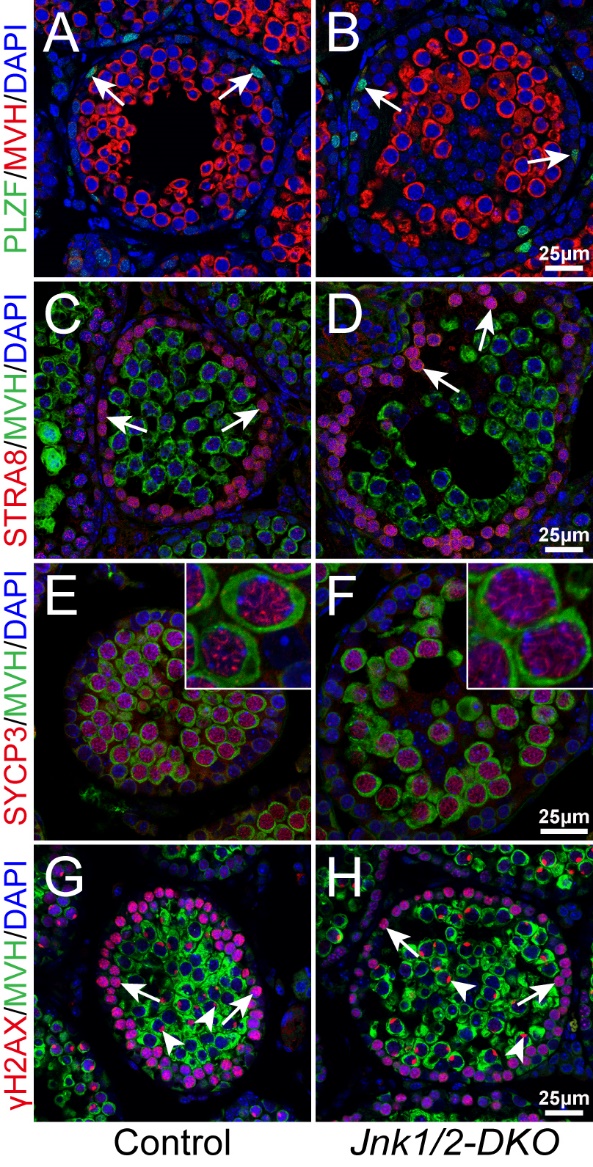
Fig. S3. The process of meiosis is not affected in *Jnk1/2-DKO* mice.

PLZF-positive undifferentiated spermatogonia (green, white arrows) in control (**A**) and *Jnk1/2-DKO* testes (**B**) at 4 weeks were detected by IF. STRA8-positive differentiating spermatogonia (red, white arrows) in control (**C**) and *Jnk1/2-DKO* testes (**D**) at 4 weeks were detected by IF. SYCP3-positive spermatocytes in control (**E**) and *Jnk1/2-DKO* testes (**F**) at 4 weeks were detected by IF. Spermatocytes with scattered (white arrows) or XY-body-enriched (white arrow heads) γH2AX in control (**G**) and *Jnk1/2-DKO* mice (**H**) at 4 weeks were detected by IF. Scale bars: 25 μm.

##
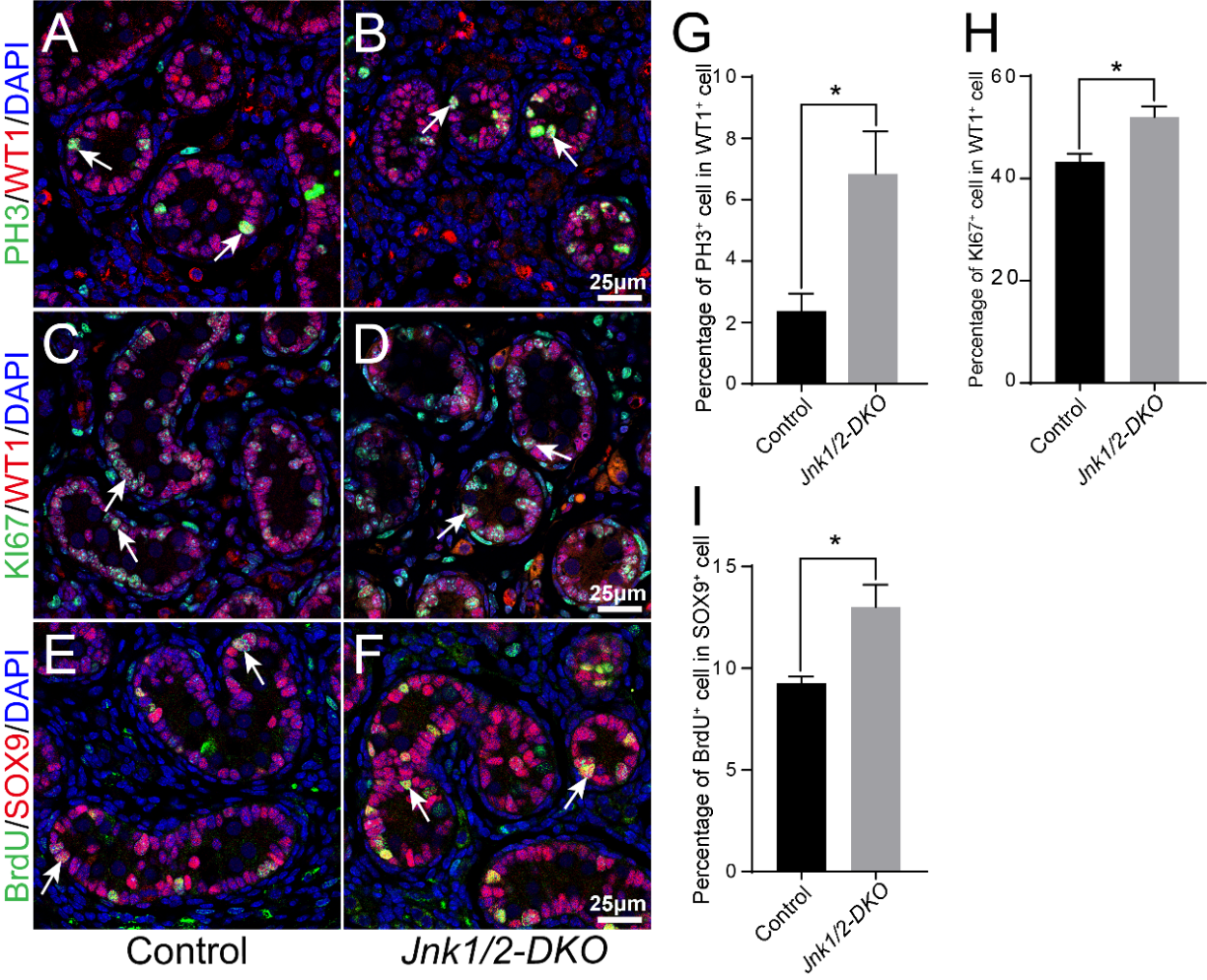
Fig. S4. The proliferation of Sertoli cells is increased in nascent *Jnk1/2-DKO* mice.

The proliferation of Sertoli cells in P0 control and *Jnk1/2-DKO* testes was examined by PH3 (**A**, **B**), KI67 (**C**, **D**), and BrdU (**E**, **F**) staining, with Sertoli cells labeled with WT1 or SOX9 (**A**-**F**, red). (**G-I**) Statistical results of proliferating Sertoli cells (double positive cells, white arrows) in the testis of nascent control and *Jnk1/2-DKO* mice. The data are presented as mean ± SEM. n = 3; *p < 0.05.


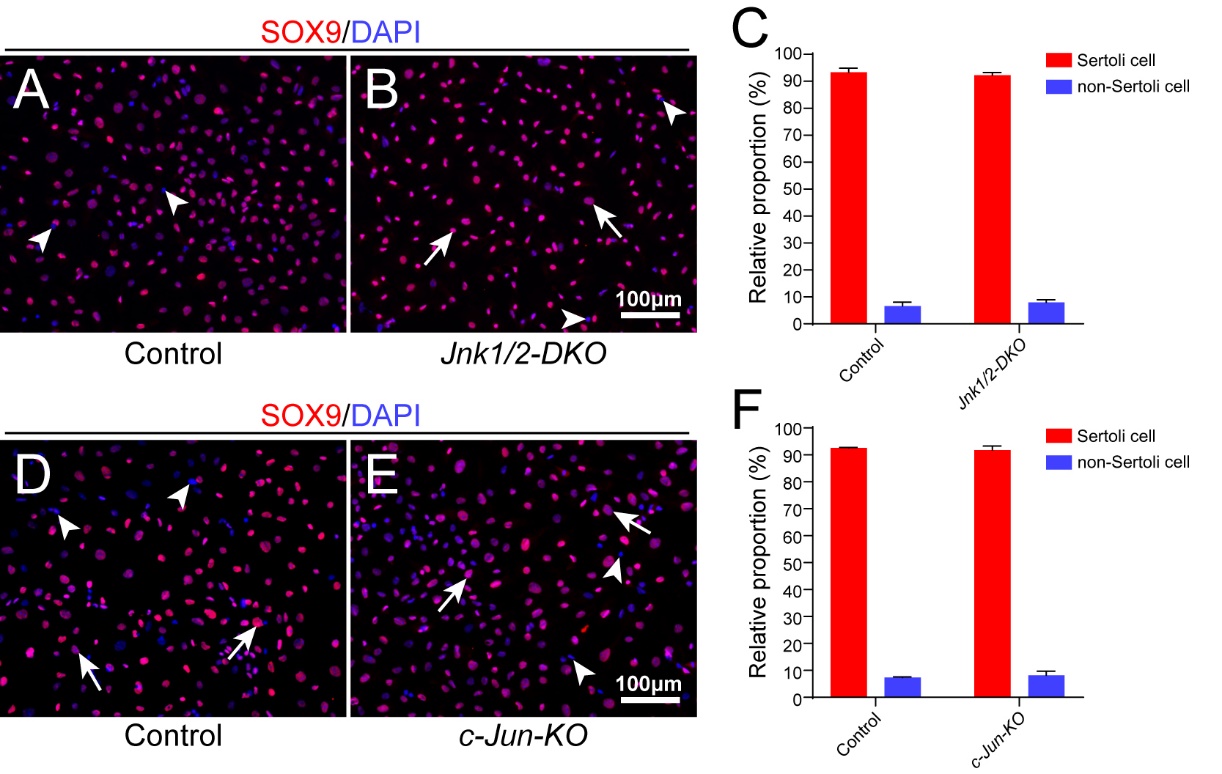


## Fig. S5. Purity of cultured primary Sertoli cells.

The purity of Sertoli cells was evaluated by immunofluorescence of SOX9. Sertoli cells isolated from control (**A**, **D**), *Jnk1/2-DKO* (**B**) and *c-Jun-KO* (**E**) were labeled with SOX9 (red), and the nuclei were stained with DAPI (blue). Arrows point to Sertoli cells, arrowheads point to non-Sertoli cells. (**C**) The relative proportion of Sertoli cells was 93.4 ± 1.4% (control) and 92.3 ± 1.0% (*Jnk1/2-DKO*) (**F**) The relative proportion of Sertoli cells was 92.6 ± 0.2% (control) and 91.8 ± 1.5% (*c-Jun-KO*). Data are presented as mean ± SEM. n = 3.

##
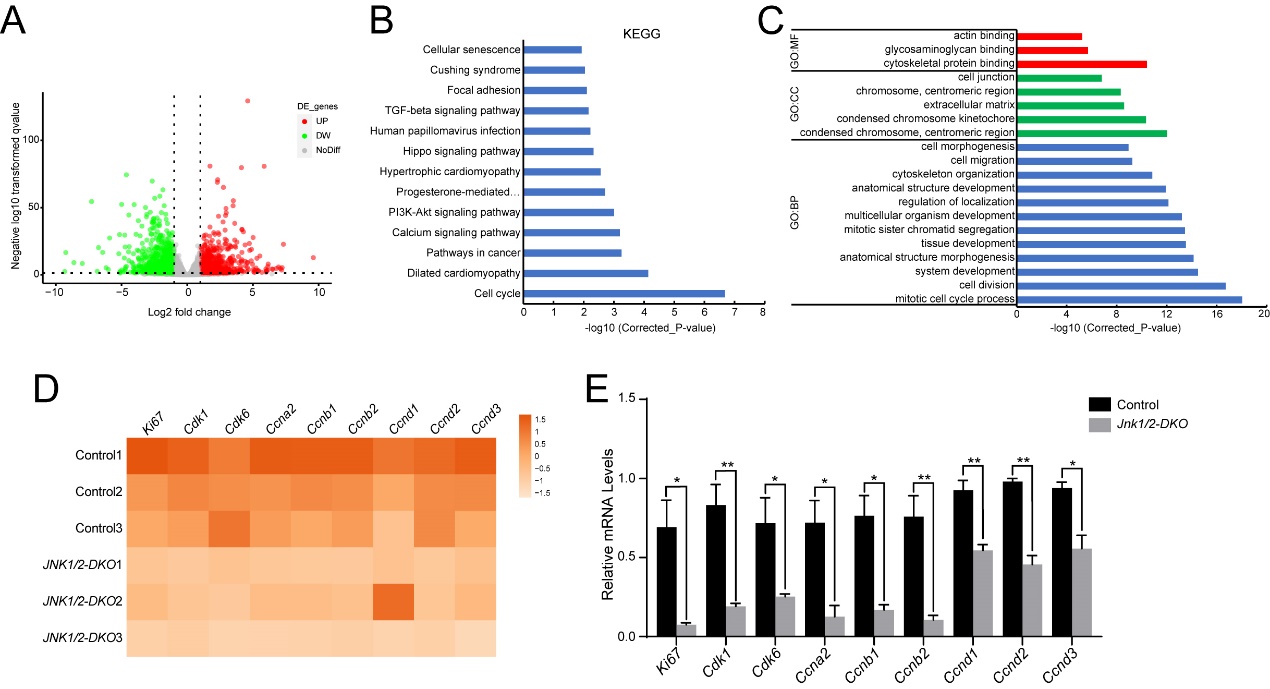
Fig. S6. Differentially expressed genes in Sertoli cells of *Jnk1/2-DKO* mice.

(**A**) Differentially expressed genes in Sertoli cells of control and *Jnk1/2-DKO* mice. (**B**) KEGG pathway analysis of the differentially expressed genes. (**C**) GO term analysis of the differentially expressed genes. (**D**) Heatmap from RNA-Seq showed that *Ki67*, *Cdk1*, *Cdk6*, *Ccna2*, *Ccnb1*, *Ccnb2*, *Ccnd1*, *Ccnd2* and *Ccnd3* were down-regulated in *Jnk1/2-DKO* Sertoli cells. (**E**) qRT-PCR results. The data are presented as mean ± SEM. n=3; *p < 0.05; **p < 0.01.

##
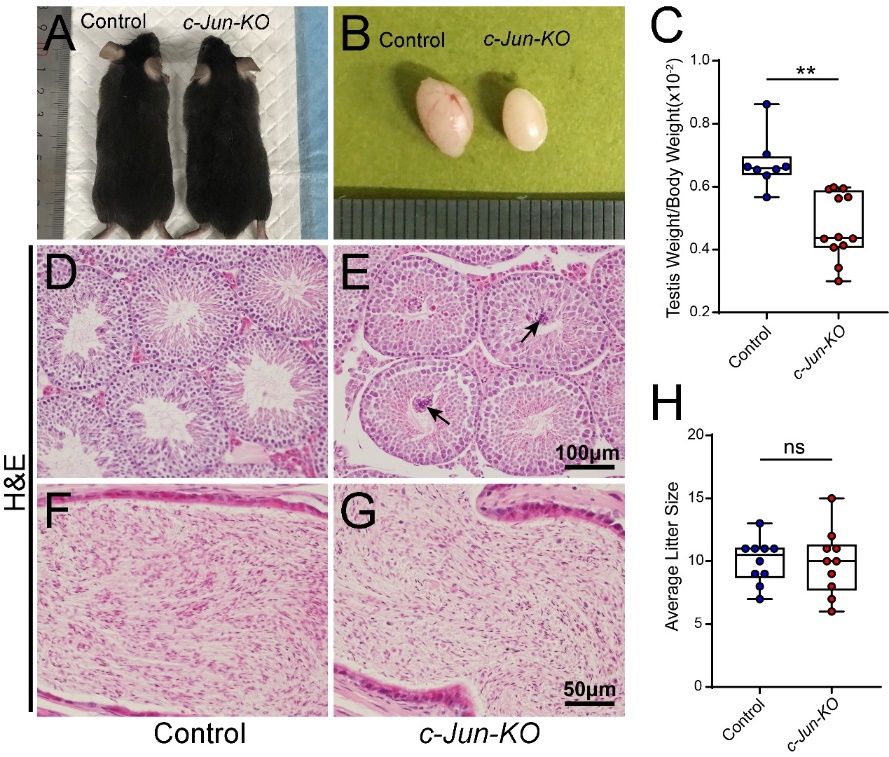
Fig. S7. No defects of germ cell development are observed in adult *c-Jun-KO* males.

(**A**) No obvious developmental defects were observed in adult *c-Jun-KO* male mice. (**B**, **C**) The size of testes in *c-Jun-KO* mice was smaller than that of control mice. (**D**-**G**) H&E staining of testicular and epididymal sections in control and *c-Jun-KO* mice. (**H**) The results of fertility test. The data are presented as mean ± SEM. n = 3; **p < 0.01; ns, not significant. Scale bars: 50 μm.


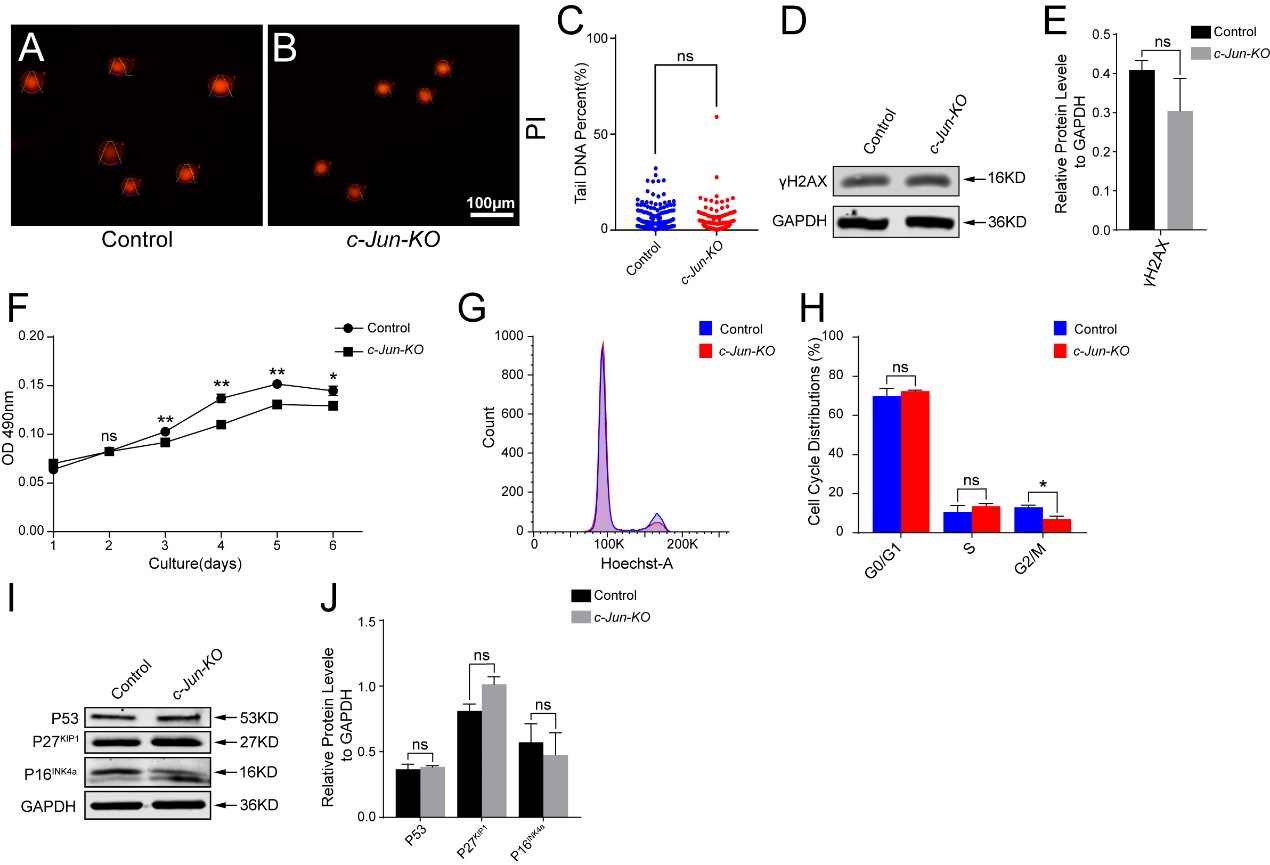


## Fig. S8. Inactivation of *c-Jun* does not cause senescence in Sertoli cells.

Representative comet assay images of control (**A**) and *c-Jun-KO* (**B**) Sertoli cells. Scale bar: 100 μm. (**C**) Quantitative analysis of tail DNA in control and *c-Jun-KO* Sertoli cells. (**D**, **E**) The protein levels of γH2AX in control and *c-Jun-KO* Sertoli cells were examined by western blotting. (**F**) Sertoli cells from control and *c-Jun-KO* mice were cultured *in vitro*, and their proliferation was assessed by MTT assay. (**G**, **H**) Cell cycle analysis of control and *c-Jun-KO* Sertoli cells was performed using flow cytometry. (**I**, **J**) The protein levels P53, P27^KIP1^ and P16^INK4a^ in control and *c-Jun-KO* Sertoli cells were examined by western blotting. The data are presented as mean ± SEM. n = 3; *p < 0.05; **p < 0.01.

## Table S1. List of antibodies used in this study.

| Antibody | Source | Catalog Code | Dilution |
| --- | --- | --- | --- |
| JNKs | CST | 9252 | 1:200 (IF), 1:1000 (WB) |
| WT1 | Abcam | ab89901 | 1:200 (IF) |
|  | Millipore | MAB4234 | 1:100(IF) |
| SOX9 | Millipore | AB5535 | 1:200 (IF), 1:500 (IHC) |
| MVH | Abcam | ab13840 | 1:400 (IF), 1:1000 (IHC) |
|  | Abcam | ab27591 | 1:200 (IF) |
| Laminin | Santa Cruz | sc-7239 | 1:200 (IF) |
| β-Catenin | Abcam | ab6306 | 1:500 (IF), 1:1000 (WB) |
| N-Cadherin | Santa Cruz | sc-7939 | 1:1000 (WB) |
| E-Cadherin | Abcam | ab76055 | 1:1000 (WB) |
| Vimentin | Abcam | ab8978 | 1:1000 (WB) |
| Vinculin | Epitomics | Jan-67 | 1:1000 (WB) |
| PLZF | R&D | AF2944 | 1:200 (IF) |
| STRA8 | Abcam | ab49405 | 1:200 (IF) |
| SYCP3 | Abcam | ab15093 | 1:200 (IF) |
| γH2AX | Millipore | 05-636 | 1:500 (IF) |
| PH3 | CST | 4620 | 1:200 (IF) |
| Ki67 | Abcam | ab15580 | 1:200 (IF) |
| p-JUN | Abcam | ab178858 | 1:1000 (WB) |
| c-JUN | Abcam | ab31419 | 1:1000 (WB) |
| JUND | Abcam | ab181615 | 1:1000 (WB) |
| P53 | Proteintech | 10442-1-AP | 1:1000 (WB) |
| P27^KIP1^ | Proteintech | 25614-1-AP | 1:1000 (WB) |
| P21^CIP1^ | Proteintech | 10355-1-AP | 1:1000 (WB) |
| P16^INK4a^ | Abcam | ab211542 | 1:1000 (WB) |
| c-MYC | Abcam | ab332072 | 1:1000 (WB) |
| E2F1 | Ruiying | RLT1442 | 1:1000 (WB) |
| GAPDH | ADI | AB1019T | 1:5000(WB) |

## Table S2. List of primers used for plasmids construction.

| Name | Primer 5′ to 3′ |
| --- | --- |
| *c-Jun*-cDNA-F | GTCGACGTCGCCACCATGACTGCAAAGATGGAAAC |
| *c-Jun*-cDNA-R | TGGTCCTTATAGTCAAACGTTTGCAACTGCTGCGTT |
| *c-Jun*-EGFP-N1-F | TTGCAAACGTTTGACTATAAGGACCACGACGGAG |
| *c-Jun*-EGFP-N1-R | GCAGTCATGGTGGCGACGTCGACTGCAGAATT |
| *c-Jun*-AA-63F | GACCTTCTCACGGCGCCCGACGTCGGGCT |
| *c-Jun*-AA-63R | CGACGTCGGGCGCCGTGAGAAGGTC |
| *c-Jun*-AA-73F | TCAAGCTGGCGGCGCCGGAGCTGGA |
| *c-Jun*-AA-73R | TCCAGCTCCGGCGCCGCCAGCTTGA |
| *c-Myc*-cDNA-F | CGACGTCGCCACCATGCCCCTCAACGTGAA |
| *c-Myc*-cDNA-R | GTCCTTATAGTCTGCACCAGAGTTTCGAAG |
| *c-Myc*-EGFP-N1-F | CTCTGGTGCAGACTATAAGGACCACGACGG |
| *c-Myc*-EGFP-N1-R | AGGGGCATGGTGGCGACGTCGACTGCAG |
| TGF-β2-promoter-F | TCTTACGCGTAATAATGGAGGTTTTTAGAA |
| TGF-β2-promoter-R | CGGGC TAGCATCACGATTCTGCCCGGAGCA |
| TGF-β2-PGL3-F | AATCGTGATGCTAGCCCGGGCTCGAGATCT |
| TGF-β2-PGL3-R | CTCCATTATTACGCGTAAGAGCTCGGTACC |
| *Jam-B*-promoter-F | ACGCGTATTTCCCCAAGGCATCTCATCCGA |
| *Jam-B*-promoter-R | CCGGGCTAGCTGATCCGCTTTGTGTCTAGT |
| *Jam-B*-PGL3-F | CGGATCAGCTAGCCCGGGCTCGAGATCT |
| *Jam-B*-PGL3-R | TTGGGGAAATACGCGTAAGAGCTCGGTACC |

## Table S3. List of primers used for qRT-PCR.

| Gene | Forward primer 5′ to 3′ | Reverse primer 5′ to 3′ |
| --- | --- | --- |
| *Jnk1* | AGCCGGCCATTTCAGAATCA | GGGATTTCTGTGGTGTGAAAACA |
| *Ki67* | ATCATTGACCGCTCCTTTAGGT | GCTCGCCTTGATGGTTCCT |
| *Cdk1* | AGAAGGTACTTACGGTGTGGT | GAGAGATTTCCCGAATTGCAGT |
| *Cdk6* | GGCGTACCCACAGAAACCATA | AGGTAAGGGCCATCTGAAAACT |
| *Ccna2* | GCCTTCACCATTCATGTGGAT | TTGCTCCGGGTAAAGAGACAG |
| *Ccnb1* | AAGGTGCCTGTGTGTGAACC | GTCAGCCCCATCATCTGCG |
| *Ccnb2* | GCCAAGAGCCATGTGACTATC | CAGAGCTGGTACTTTGGTGTTC |
| *Ccnd1* | GCGTACCCTGACACCAATCTC | ACTTGAAGTAAGATACGGAGGG |
| *Ccnd2* | GAGTGGGAACTGGTAGTGT | CGCACAGAGCGATGAAGGT |
| *Ccnd3* | CATGGCAGTTGCGGGAGT | TTTTGACCAAAGCCTGCCG |
| *c-Jun* | CCTTCTACGACGATGCCCTC | GGTTCAAGGTCATGCTCTGTTT |
| *JunD* | GGCGGGATTGAAACCAGGG | AGCCCGTTGGACTGGATGA |
| *TGFβ2* | TCGACATGGATCAGTTTATGCG | CCCTGGTACTGTTGTAGATGGA |
| *TGFβ3* | CAGGCCAGGGTAGTCAGAG | ATTTCCAGCCTAGATCCTGCC |
| *Jam-B* | GTGCCCACTTCTGTTATGACTG | TTCCCTAGCAAACTTGTGCCA |
| *c-Myc* | TTGGAAACCCCGCAGACAG | GCTGTACGGAGTCGTAGTCG |
| *Skp1* | ATGCCTACGATAAAGTTGCAGAG | TCCATTCCCAAATCTTCCAGC |
| *Skp2* | ATGGACTGCTCTCAAACCTCG | CCTGGAAAGTTCTCCCGACTAA |
| *Cks1b* | TATTCGGACAAATACGACGACG | GGTTCCTCCATTCAGATTCAGAC |
| *E2f1* | CAGAACCTATGGCTAGGGAGT | GATCCAGCCTCCGTTTCACC |
| *Gapdh* | TTGTCTCCTGCGACTTCAACA | TTGTCTCCTGCGACTTCAACA |
